# Pentylenetetrazole-Induced Seizures Are Increased after Kindling, Exhibiting Vitamin-Responsive Correlations to the Post-Seizures Behavior, Amino Acids Metabolism and Key Metabolic Regulators in the Rat Brain

**DOI:** 10.3390/ijms241512405

**Published:** 2023-08-03

**Authors:** Vasily A. Aleshin, Anastasia V. Graf, Artem V. Artiukhov, Alexander L. Ksenofontov, Lev G. Zavileyskiy, Maria V. Maslova, Victoria I. Bunik

**Affiliations:** 1 A.N. Belozersky Institute of Physicochemical Biology, Lomonosov Moscow State University, 119991 Moscow, Russia; aleshinvasily@gmail.com (V.A.A.);; 2 Department of Biochemistry, Sechenov University, Trubetskaya, 8, Bld. 2, 119991 Moscow, Russia; 3 Faculty of Biology, Lomonosov Moscow State University, 119234 Moscow, Russia; 4 Faculty of Nano-, Bio-, Informational, Cognitive and Socio-Humanistic Sciences and Technologies at Moscow Institute of Physics and Technology, Maximova Street 4, 123098 Moscow, Russia; 5 Faculty of Bioengineering and Bioinformatics, Lomonosov Moscow State University, 119991 Moscow, Russia

**Keywords:** pentylenetetrazole, vitamin B1, vitamin B6, p53, sirtuin, acetylation, succinylation, 2-oxoglutarate dehydrogenase (alpha-ketoglutarate dehydrogenase), NAD^+^

## Abstract

Epilepsy is characterized by recurrent seizures due to a perturbed balance between glutamate and GABA neurotransmission. Our goal is to reveal the molecular mechanisms of the changes upon repeated challenges of this balance, suggesting knowledge-based neuroprotection. To address this goal, a set of metabolic indicators in the post-seizure rat brain cortex is compared before and after pharmacological kindling with pentylenetetrazole (PTZ). Vitamins B1 and B6 supporting energy and neurotransmitter metabolism are studied as neuroprotectors. PTZ kindling increases the seizure severity (1.3 fold, *p* < 0.01), elevating post-seizure rearings (1.5 fold, *p* = 0.03) and steps out of the walls (2 fold, *p* = 0.01). In the kindled vs. non-kindled rats, the post-seizure p53 level is increased 1.3 fold (*p* = 0.03), reciprocating a 1.4-fold (*p* = 0.02) decrease in the activity of 2-oxoglutarate dehydrogenase complex (OGDHC) controlling the glutamate degradation. Further, decreased expression of deacylases SIRT3 (1.4 fold, *p* = 0.01) and SIRT5 (1.5 fold, *p* = 0.01) reciprocates increased acetylation of 15 kDa proteins 1.5 fold (*p* < 0.01). Finally, the kindling abrogates the stress response to multiple saline injections in the control animals, manifested in the increased activities of the pyruvate dehydrogenase complex, malic enzyme, glutamine synthetase and decreased malate dehydrogenase activity. Post-seizure animals demonstrate correlations of p53 expression to the levels of glutamate (r = 0.79, *p* = 0.05). The correlations of the seizure severity and duration to the levels of GABA (r = 0.59, *p* = 0.05) and glutamate dehydrogenase activity (r = 0.58, *p* = 0.02), respectively, are substituted by the correlation of the seizure latency with the OGDHC activity (r = 0.69, *p* < 0.01) after the vitamins administration, testifying to the vitamins-dependent impact of the kindling on glutamate/GABA metabolism. The vitamins also abrogate the correlations of behavioral parameters with seizure duration (r 0.53–0.59, *p* < 0.03). Thus, increased seizures and modified post-seizure behavior in rats after PTZ kindling are associated with multiple changes in the vitamin-dependent brain metabolism of amino acids, linked to key metabolic regulators: p53, OGDHC, SIRT3 and SIRT5.

## 1. Introduction

Epilepsy is a common, predominantly polygenic, disease characterized by recurrent seizures due to a perturbed balance between glutamate and GABA neurotransmission [1,2,3,4,5]. It is known that repeated sub-convulsive challenges of this balance cumulate into heightened seizure activity. This process, known as kindling, may be modelled by repeated administration of seizures-inducing chemicals [6,7,8,9], or electrical stimulation of certain brain area [10,11]. An advantage of the chemical kindling is that the affected protein targets are usually well defined. In particular, the imbalance between excitatory and inhibitory neurotransmission due to administration of the widely used epileptogenic compound pentylenetetrazole (PTZ) [12,13,14,15] involves its binding to the GABA-A receptor [14].

Independent studies point to the kindling-induced perturbations in brain neurotransmission, followed by neuronal damage. In particular, kindling is associated with cognitive dysfunctions due to the neuronal damage in the hippocampus [16]. Kindling also increases the expression and membrane translocation of rho kinase (ROCK II), whose inhibition decreases sensitivity to epileptogenic factors, probably due to the kinase association with activation of glutamatergic signaling [17]. In many brain regions, density of A1 adenosine receptors, NMDA and kainate glutamate receptors, as well as expression of heat shock proteins is increased by repeated PTZ injections more than by a single PTZ injection [18,19,20].

Perturbed glutamatergic neurotransmission plays a crucial role in increased generation of free radical species and neuronal cell death in the PTZ-kindled rats [21]. In various areas of the brain of rats and mice, including cerebral cortex, PTZ kindling increases oxidative stress. This is evident from elevated levels of H_2_O_2_, O_2_^−•^, nitrite, malonic aldehyde, 4-hydroxynonenal, accompanied by increased activities of the antioxidant enzymes superoxide dismutase and catalase and decreased levels of reduced glutathione (GSH) [22,23,24,25,26]. Remarkably, brain GSH level correlates negatively with the Racine seizure score and positively—with the locomotor activity and recognition memory after seizures [22].

Despite cerebral metabolism is tightly linked to the kindling-affected redox balance, neurotransmission and damage response, the kindling-associated metabolic changes are characterized in a rather fragmentary way. Compared to a single PTZ-induced seizure, the PTZ kindling is known to elevate the brain activities of alkaline phosphatase, glutamate-oxaloacetate transaminase (GOT) and lactate dehydrogenase (LDH), decreasing that of creatine kinase [27]. In addition, ATP hydrolysis in synaptosomes from the kindled animals is increased, compared to the single PTZ-induced seizures [28]. Different changes in post-translational modifications (PTM) of the brain proteins after the seizures induced without and after the PTZ kindling are revealed in our recent work employing mass spectrometry [29], suggesting the regulation of metabolic proteins by PTM to be involved in the kindling process. In particular, compared to the control brain of animals without seizures, the seizures after kindling are associated with decreased levels of malonylation of dihydrolipoamide acetyltrasferase (ODP2) or glutarylation of hexokinase, that is not observed after the seizures without the kindling. In contrast, the acetylation and glutarylation of the brain lactate dehydrogenase (LDHA) is not changed after the kindling, but is decreased vs. the control level after single seizures [29]. The different responses of different proteins to the specific pathological states, such as those observed post-seizures in the kindled or non-kindled animals, suggest biphasic metabolic regulation by the PTM in response to increasing perturbation induced by the kindling. That is, the acylations of lactate dehydrogenase are involved into the brain response to single seizures, returning to the control levels after seizures in the kindled animals. In contrast, acylations of ODP2 and hexokinase respond to seizures only in the kindled animals, remaining unchanged vs. control state after single seizures. It is also worth noting that the PTM level is changed in the metabolic proteins involved in transformation of a key branch point metabolite pyruvate, connecting glycolysis to mitochondrial energy production. Thus, PTM of these proteins may participate in the kindling-associated reprogramming of metabolic fluxes through these pathways. Mutual regulation of these fluxes depends on transcriptional factor p53, a master regulator of energy metabolism, that is known to control perturbations of neuronal activity, particularly in epileptic seizures and upon kindling [30,31,32,33,34]. In its turn, such PTM as phosphorylation strongly regulates the function and stability of p53 [35,36,37,38]. However, specific significance of the p53 phosphorylation in epilepsy is not studied as extensively as it is in cancer [35,37].

In humans, epileptic seizures arise upon disturbed metabolism of vitamins B1 or B6 [39,40,41,42]. In seizures-inducing encephalopathies, administration of B1 and B6 vitamins may be neuroprotective [43], while vitamin B1 and glucose are recommended in hypoglycemic patients with epilepsy [44]. These data stress the medical significance of the brain metabolism dependent on B1 and B6 vitamins. However, the molecular mechanisms of their action in epileptic seizures are not systematically studied.

The goal of the current work is to characterize the link between the central brain metabolism and pathophysiology in a rat model of PTZ-induced seizures, revealing the impact of the PTZ kindling on the brain metabolism. Our characterization of the seizures, behavior and ECG is therefore accompanied by simultaneous assessment of a number of key biochemical parameters of the brain, that may be relevant for the pathogenesis. These parameters include the levels of p53 and its regulatory phosphorylation, the functions of enzymes participating in the brain metabolism of neurotransmitters and energy production, the levels of the NAD^+^-dependent deacylases linked to the functions of these enzymes, and the levels of metabolites of the considered pathways. Potential involvement in the PTZ kindling of epigenetic mechanisms through different types of acylation of histones [45] is assessed by estimation of the kindling role in the acetylation and succinylation of the brain proteins of the low molecular masses corresponding to histones. The acylations of the brain proteins is also detected along with the protein expression of the corresponding deacylases SIRT3 and SIRT5. In view of the critical dependence of the brain energy and neurotransmitters metabolism on vitamins B1 and B6, both exhibiting neurotropic action underlying potential therapeutic significance in neurological disorders including epilepsy [46,47,48], we also evaluate the metabolic and physiological effects of supplementation of vitamins B1 and B6 on the seizures without or after the PTZ kindling.

## 2. Results

**Figure 1:**
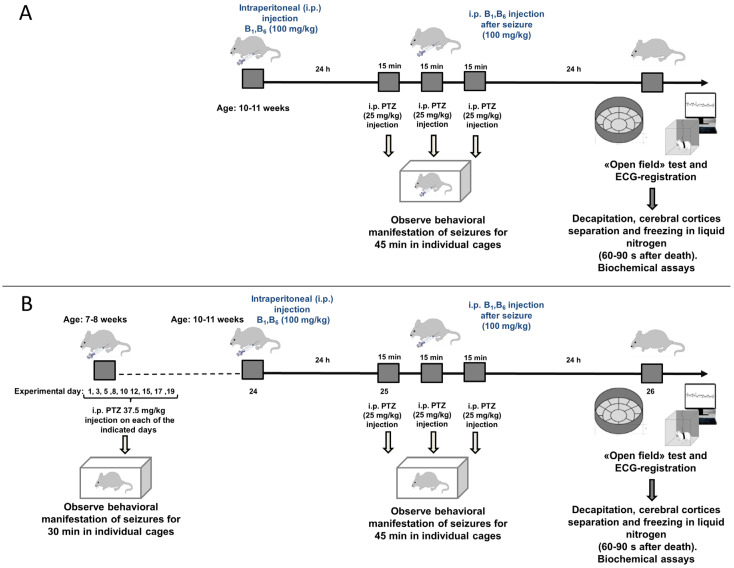
**The flowcharts of the two models of PTZ-induced seizures.** (**A**)—The flowchart for the single episode of epileptic seizures induced by PTZ (SA group). The seizures are induced by up to three injections of 25 mg/kg PTZ, depending on the development of the seizure. Next day the “open field” test and ECG are performed, after that the rats are sacrificed, the cerebral cortices extracted and frozen in liquid nitrogen. (**B**)—The flowchart for the PTZ kindling (SB group). The seizures according to the protocol described in (**A**), are induced after 9 injections of 37.5 mg/kg PTZ on the indicated days within 20 days. In both protocols, the vitamins B1 and B6 (100 mg/kg each) are injected 24 h before the seizure induction and 45 min after the first PTZ injection. The effects of injections without the substances are tested on the control animals (CA or CB) receiving saline (0.9% NaCl) or saline and vitamins at the times and in the volumes, equal to those of PTZ and/or vitamins, according to the group assignment.

To reveal the molecular mechanisms of PTZ kindling and associated action of the vitamins B1 and B6, the seizures and post-seizures parameters are characterized in the two experimental settings, shown in Figure 1.

The protocol in Figure 1A (further the animal group SA throughout the text) shows the induction of a single seizures by PTZ, whereas the protocol in Figure 1B (further the animal group SB throughout the text) shows the induction of the seizures after the PTZ kindling. To compare the seizures-exposed animals at the same age (10–11 weeks), the kindling procedure starts at the age of 7–8 weeks.

As the treatment conditions, required for the PTZ kindling, include multiple injections, the injections-associated stress may have physiological and/or biochemical effects per se. Hence, to discriminate the effects of the PTZ kindling and those of the associated injections, the differences between the experimental SB and SA groups are analyzed in view of the potential stress effects in the corresponding control groups receiving the injections of saline without the groups-assigned substances according to the protocol B (CB group, Figure 1B) or protocol A (CA group, Figure 1A).

The further details of animal experiments are described in Methods.

### 2.1. PTZ Kindling Increases Severety of Seizures

**Figure 2:**
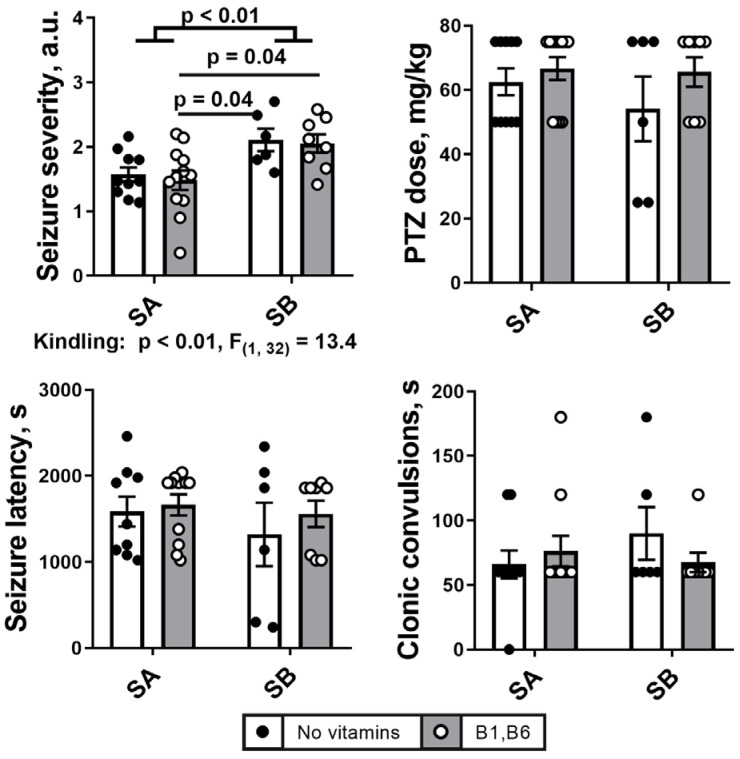
**Parameters of the PTZ-induced seizures observed without and after the PTZ kindling.** Seizures (S) are induced according to the two protocols shown in Figure 1: SA defines the animals treated by the protocol in Figure 1A, i.e., without the PTZ kindling (n = 10 without vitamins; n = 12 with vitamins); SB defines the animals subjected to the PTZ kindling according to the protocol in Figure 1B (n = 6 without vitamins; n = 8 with vitamins). The data on animals receiving vitamins B1 and B6 are represented by grey bars, as shown in the legend. The seizures parameters are characterized as described in “Materials and methods”. Statistically significant (*p* < 0.05) differences between the experimental groups, determined using the two-way ANOVA with Tukey’s post hoc test, are shown on the graphs. Further, significant factors, determined by the two-way ANOVA, and their statistics, are indicated under the graphs.

Sensitivity of the male rats to the seizure inducer PTZ is characterized by an average PTZ dose required to induce seizures, and pathophysiological parameters of the seizures, shown in Figure 2. Comparison of the two protocols of the seizure induction by PTZ (Figure 1) indicates that the seizure severity score is significantly lower in the single seizure model (group SA), compared to the seizures after PTZ kindling (group SB). The scores are 1.6 ± 0.1 and 2.1 ± 0.2 mg/kg, for SA and SB, respectively (Figure 2). The difference is observed between the combined groups of animals, i.e., both with and without vitamin administration, although the significance is more pronounced in the vitamin-treated than non-treated animals. Otherwise, the administration of vitamins B1 and B6 24 h before the induction of seizures by PTZ does not significantly affect the parameters of the seizures, either without or with the PTZ kindling.

Other parameters of seizures do not show statistically significant effects of the PTZ kindling, yet the observed differences in the three indicated parameters, i.e., the seizure latency, average PTZ doses to induce seizures and the duration of clonic seizures exhibit consistent changes. As seen in Figure 2, after the PTZ kindling, the PTZ dose (62.5 ± 4 mg/kg in SA group vs. 54.2 ± 10 mg/kg in SB group) and seizure latency (1587.0 ± 174.1 s in SA group vs. 1320.0 ± 369.2 s in SB group) decrease, while the convulsions time increases (66.0 ± 10.8 s in SA group vs. 90.0 ± 20.5 s in SB group).

### 2.2. Effects of the PTZ Kindling and the Administration of Vitamins B1 and B6 on Behavioral and ECG Parameters after the Seizures

**Figure 3:**
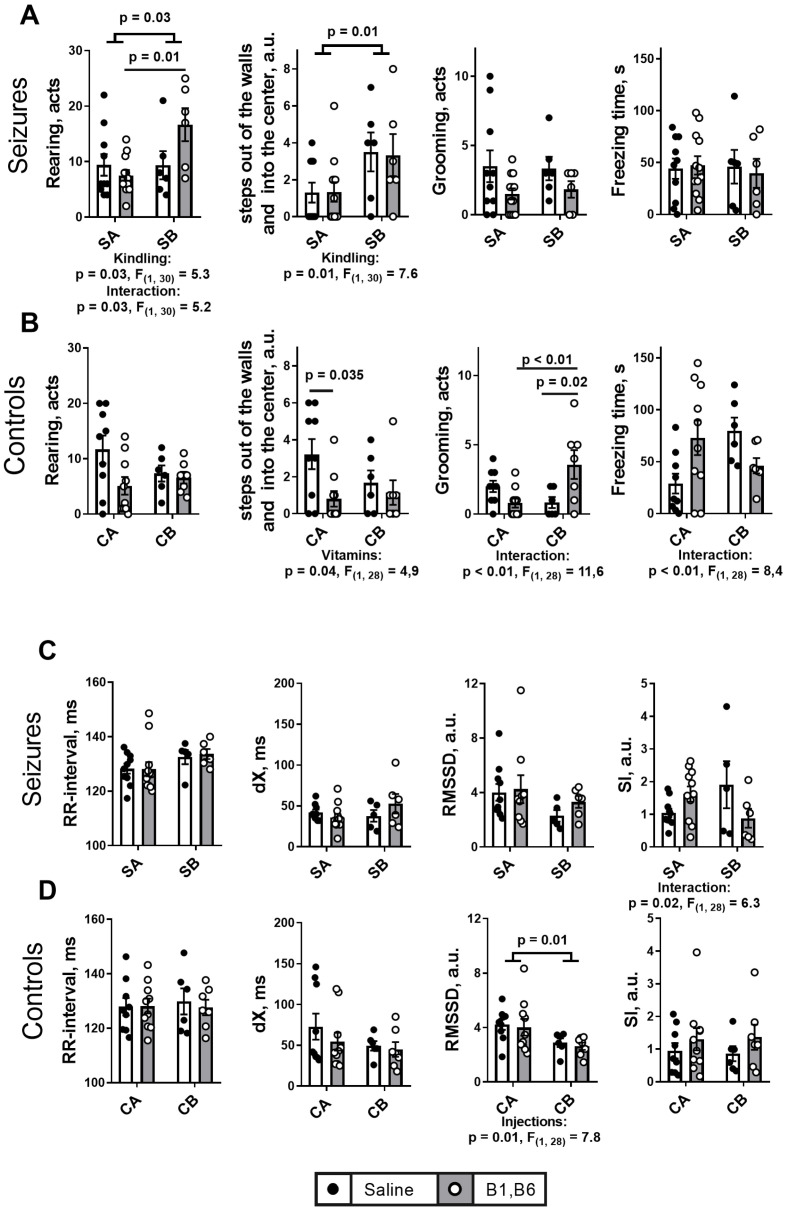
**Effects of the PTZ kindling and vitamins on the behavioral (A,B) and ECG (C,D) parameters of rats after seizures, in comparison to the effects of the corresponding injections of physiological solution in the control animals.** Seizures (S) are induced according to the two protocols shown in Figure 1: SA defines the animals treated by the protocol in Figure 1A, i.e., without the PTZ kindling (n = 10 without vitamins; n = 12 with vitamins); SB defines the animals subjected to the PTZ kindling according to the protocol in Figure 1B (n = 6 without vitamins; n = 6 with vitamins). In the control (C) animals, CA defines those receiving saline instead of vitamins and/or PTZ according to the protocol in Figure 1A (n = 9 without vitamins; n = 10 with vitamins); CB defines the analogous control animals additionally subjected to the repetitive injections of saline instead of PTZ according to the protocol in Figure 1B (n = 6 without vitamins; n = 7 with vitamins). The assayed parameters are indicated on the Y axes. Statistically significant (*p* < 0.05) differences between the experimental groups, determined using the two-way ANOVA with Tukey’s post hoc test, are shown on the graphs. Further, significant factors, determined by the two-way ANOVA, and their statistics, are indicated under the graphs.

Behavioral parameters presented in Figure 3A show that the PTZ kindling significantly increases the number of rearing acts, that is more pronounced in the animals receiving vitamins, and the steps out of the wall. These effects are specific to the PTZ kindling, as they are absent in the control animals (Figure 3B). While the control animals do not show any significant behavioral effects of the multiple injections mimicking the kindling procedure, they exhibit more reactivity to the vitamins (manifested in the steps out of the wall), whose action may also interact with the injections (manifested in the grooming acts and freezing time). These effects of the vitamins in the control animals are absent in the PTZ-kindled animals. Other assessed behavioral parameters, showing no significant effects, are presented in Supplementary Figure S6.

ECG parameters do not reveal any significant differences between the groups as a result of PTZ kindling (Figure 3C). However, in the control animals the multiple injections in the group CB decrease the relaxation index RMSSD, compared to the group CA (Figure 3D). The effect is not observed in the seizure groups.

### 2.3. PTZ Kindling Increases Expression of Transcriptional Factor p53 in the Brain

**Figure 4:**
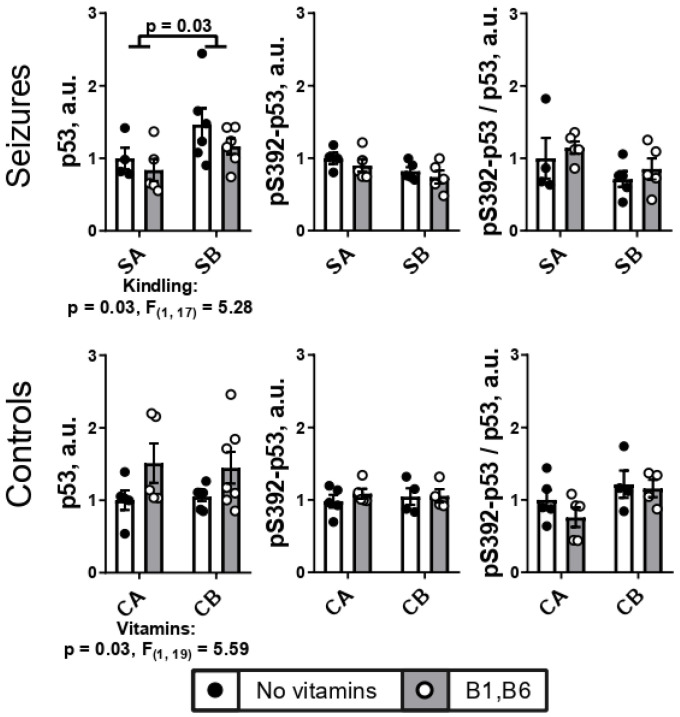
**Effects of the PTZ kindling and vitamin administration on the brain expression of p53 protein and its phosphorylation after seizures, in comparison to the effects of the corresponding injections in the control rats.** Seizures (S) are induced according to the two protocols shown in Figure 1: SA defines the animals treated by the protocol in Figure 1A, i.e., without the PTZ kindling (n = 4 without vitamins; n = 5 with vitamins); SB defines the animals subjected to the PTZ kindling according to the protocol in Figure 1B (n = 6 without vitamins; n = 6 with vitamins). In the control (C) animals, CA defines those receiving saline instead of vitamins and/or PTZ according to the protocol in Figure 1A (n = 5 without vitamins; n = 5 with vitamins); CB defines the analogous control animals additionally subjected to the repetitive injections of saline instead of PTZ according to the protocol in Figure 1B (n = 6 without vitamins; n = 7 with vitamins). Statistically significant (*p* < 0.05) differences between the experimental groups, determined using the two-way ANOVA with Tukey’s post hoc test, are shown on the graphs. Further, significant factors, determined by the two-way ANOVA, and their statistics, are indicated under the graphs. The levels of p53 or its phosphorylated at Ser392 form (pS392) are normalized to total protein in the lane and shown as a ratio to the levels in the comparison groups without vitamins (CA or SA) (see “Materials and Methods”). The original images of the blots are provided as Supplementary Figure S5.

Upregulated in response to different types of cellular stresses, transcription factor p53 is known to have an important dual role in the neuronal damage, particularly the damage induced by seizures [32]. Figure 4 shows that the seizures after the PTZ kindling are associated with increased expression of the brain p53, compared to the animals undergoing seizures without the kindling. In average, an 1.3-fold increase is observed after the kindling. No significant effects of the kindling and vitamins are found when the Ser392-phosphorylated p53 is assessed. However, the changes in the p53 expression and phosphorylation in each animal of the studied groups appear to be roughly proportional, as the ratio of the p53 phosphorylation at Ser392 to the p53 expression, averaged after determination in each animal, is not changed by seizures with or without kindling (Figure 4).

The role of the repeated injections of PTZ in the p53 upregulation is confirmed by no changes in the p53 expression due to the different injection protocols in the control groups CA and CB. Interestingly, in these control animals, administration of the vitamins is a factor upregulating the p53 expression, in contrast to the seizures-exposed animals. The p53 phosphorylation or its ratio to the p53 expression are not significantly affected by the kindling, or injections, or vitamins (Figure 4).

Thus, p53 expression is increased after seizures in the PTZ-kindled vs. non-kindled animals, that is not observed after the multiple injections of saline, mimicking the kindling procedure.

As p53 is a master regulator of energy metabolism greatly relying on the B1- and B6-dependent metabolic pathways [49] and the TCA-cycle-limiting OGDHC in particular [50], we next examined a representative set of the B1,B6-dependent and related activities of the brain homogenate enzymes, working at the cross-roads of the energy production and glutamate/GABA biosynthesis.

### 2.4. Effect of PTZ Kindling and Administration of Vitamins B1 and B6 on the Activities of the B1- and B6-Dependent and Related Enzymes in the Animal Brain after Seizures

**Figure 5:**
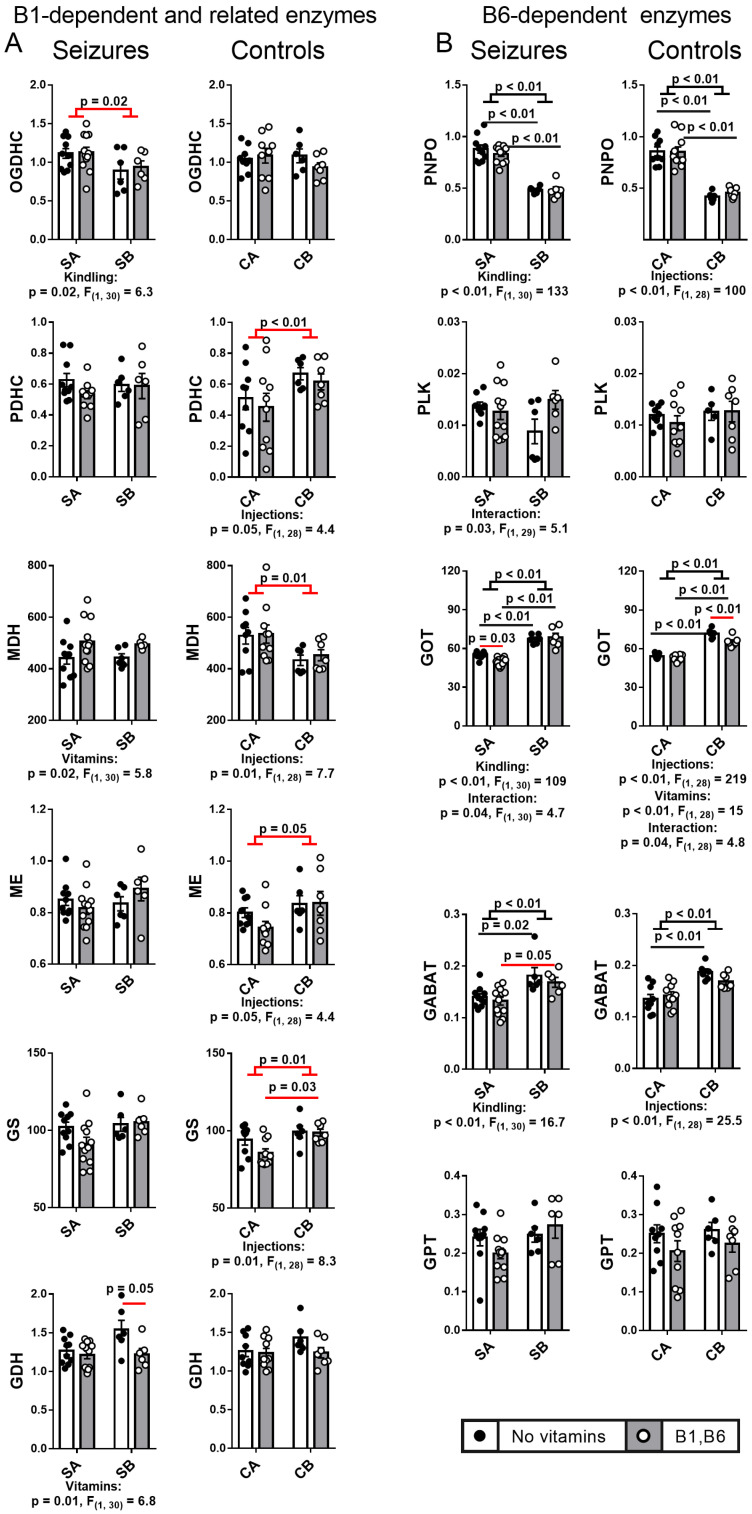
**Effects of the PTZ kindling and vitamin administration on the activities of cerebral cortex enzymes related to vitamin B1** (**A**) **and B6** (**B**)**, after seizures, in comparison to the effects of the corresponding injections in the control rats.** Seizures (S) are induced according to the two protocols shown in Figure 1: SA defines the animals treated by the protocol in Figure 1A, i.e., without the PTZ kindling (n = 10 without vitamins; n = 12 with vitamins); SB defines the animals subjected to the PTZ kindling according to the protocol in Figure 1B (n = 6 without vitamins; n = 6 with vitamins). In the control (C) animals, CA defines those receiving saline instead of vitamins and/or PTZ according to the protocol in Figure 1A (n = 9 without vitamins; n = 10 with vitamins); CB defines the analogous control animals additionally subjected to the repetitive injections of saline instead of PTZ according to the protocol in Figure 1B (n = 6 without vitamins; n = 7 with vitamins). Statistically significant (*p* < 0.05) differences between the experimental groups, determined using the two-way ANOVA with Tukey’s post hoc test, are shown on the graphs. Further, significant factors, determined by the two-way ANOVA, and their statistics, are indicated under the graphs. Different effects in the control and seizures groups are indicated with red lines. The assayed parameters are indicated on the Y axes. GDH—glutamate dehydrogenase, GS—glutamine synthetase, OGDHC—2-oxoglutarate dehydrogenase complex, PDHC—pyruvate dehydrogenase complex, ME—malic enzyme, MDH—malate dehydrogenase, GOT—glutamate oxaloacetate transaminase, GABAT—GABA transaminase, GPT—glutamate pyruvate transaminase, PLK—pyridoxal kinase, and PNPO—pyridoxamine 5′-phosphate oxidase.

Complex changes in the activities of central metabolic brain enzymes are observed in the employed experimental models. The presentation of the data in Figure 5 helps discriminating the after-seizures effects of the PTZ kindling and the stress response to multiple injections associated with the kindling model.

Independent of the vitamins, the PTZ kindling causes a seizure-induced decrease in the brain OGDHC activity, that is not observed in the control animals (Figure 5), reciprocating the effect of the PTZ kindling on p53 (Figure 4). In contrast, in response to the equivalent number of saline injections, mimicking the PTZ kindling and seizure induction procedures, the control (without seizures) animals demonstrate a strong coupled effect of the injections on the brain activities of PDHC, MDH, ME and GS. That is, independent of vitamins administration, the PDHC, ME and GS activities increase, while the MDH activity decreases (Figure 5, B1-dependent and related enzymes in Control panel). This stress response of the brain enzymes is not observed in the animals after seizures.

Thus, the brain effects of the PTZ kindling vs. single PTZ-induced seizure include not only a decrease in the brain activity of OGDHC, but also attenuation of the brain stress response, inherent in the control animals.

### 2.5. Effect of PTZ Kindling and Administration of Vitamins B1 and B6 on the Brain Protein Acylation System

Perturbed function of the producers of acetyl-CoA and succinyl-CoA, i.e., PDHC and OGDHC, may be manifested in the protein acylation [51,52]. Hence, we studied how PTZ kindling would affect expression of the brain components of the acylation system.

#### 2.5.1. Brain Protein Acetylation

**Figure 6:**
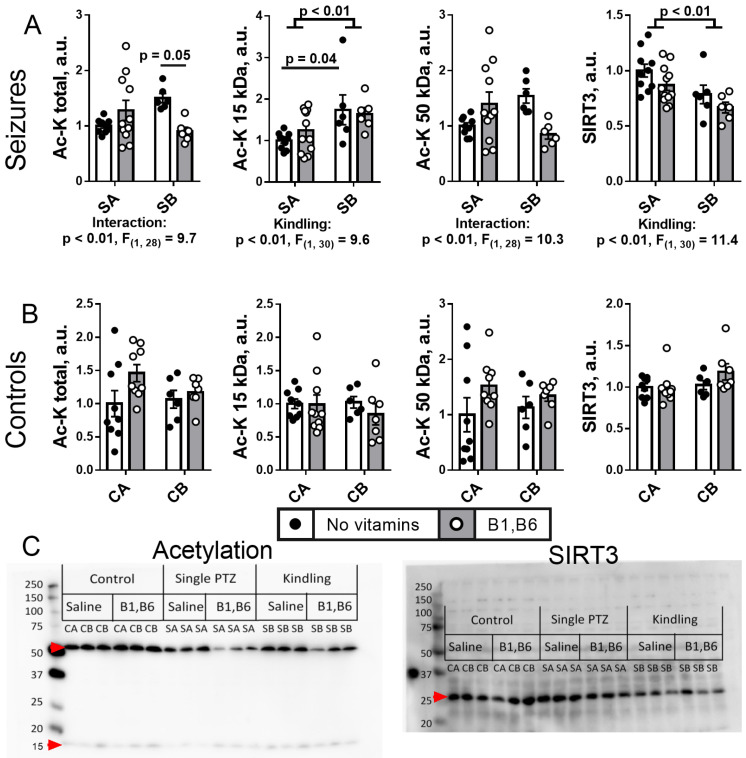
**Effects of the PTZ kindling and vitamins administration on the brain protein acetylation and mitochondrial deacetylase Sirtuin 3 after seizures** (**A**)**, in comparison to the effects of the corresponding injections in the control rats** (**B**)**. Representative images of the blots are shown in** (**C**)**.** Seizures (S) are induced according to the two protocols shown in Figure 1: SA defines the animals treated by the protocol in Figure 1A, i.e., without the PTZ kindling (n = 10 without vitamins; n = 12 with vitamins); SB defines the animals subjected to the PTZ kindling according to the protocol in Figure 1B (n = 6 without vitamins; n = 6 with vitamins). In the control (C) animals, CA defines those receiving saline instead of vitamins and/or PTZ according to the protocol in Figure 1A (n = 9 without vitamins; n = 10 with vitamins); CB defines the analogous control animals additionally subjected to the repetitive injections of saline instead of PTZ according to the protocol in Figure 1B (n = 6 without vitamins; n = 7 with vitamins). Statistically significant (*p* < 0.05) differences between the experimental groups, determined using the two-way ANOVA with Tukey’s post hoc test, are shown on the graphs. Besides, significant factors, determined by the two-way ANOVA, and their statistics, are indicated under the graphs. The assayed parameters are indicated on the Y axes. The amount of acetylated protein lysine residues and the level of SIRT3 are normalized to total protein and are shown to the levels in the comparison groups without vitamins (CA or SA) (see “Materials and Methods”). The main bands are quantified in addition to the total acylation levels. The original images of the blots are provided as Supplementary Figures S1 and S2.

The protein acetylation system is affected by kindling compared to single seizures, as can be seen from Figure 6A, while no effect of multiple injections or vitamins is observed without seizures (Figure 6B). Of the two most expressed bands of acetylated proteins (Figure 6C) with the apparent masses of 50 and 15 kDa, inherent in tubulins and histones, respectively, the kindling predominantly influences acetylation of 15 kDa fraction including histones (*p* < 0.01 of “kindling” ANOVA factor). Acetylation of these proteins is increased by kindling (Figure 6A). The effect is more pronounced in the animal groups without administration of vitamins (*p* = 0.04) than after the vitamins administration (*p* > 0.05). Total acetylation of proteins significantly (*p* = 0.05) decreases upon vitamins administration to kindled rats (Figure 6A), revealing an interaction between the kindling and vitamins factors of ANOVA (*p* < 0.01). A higher histone acetylation after kindling is observed simultaneously with a decreased (*p* < 0.01) level of the mitochondrial deacetylase sirtuin 3 (Figure 6A), that is also reported to deacetylate histones in the nucleus [53,54].

#### 2.5.2. Brain Protein Succinylation

**Figure 7:**
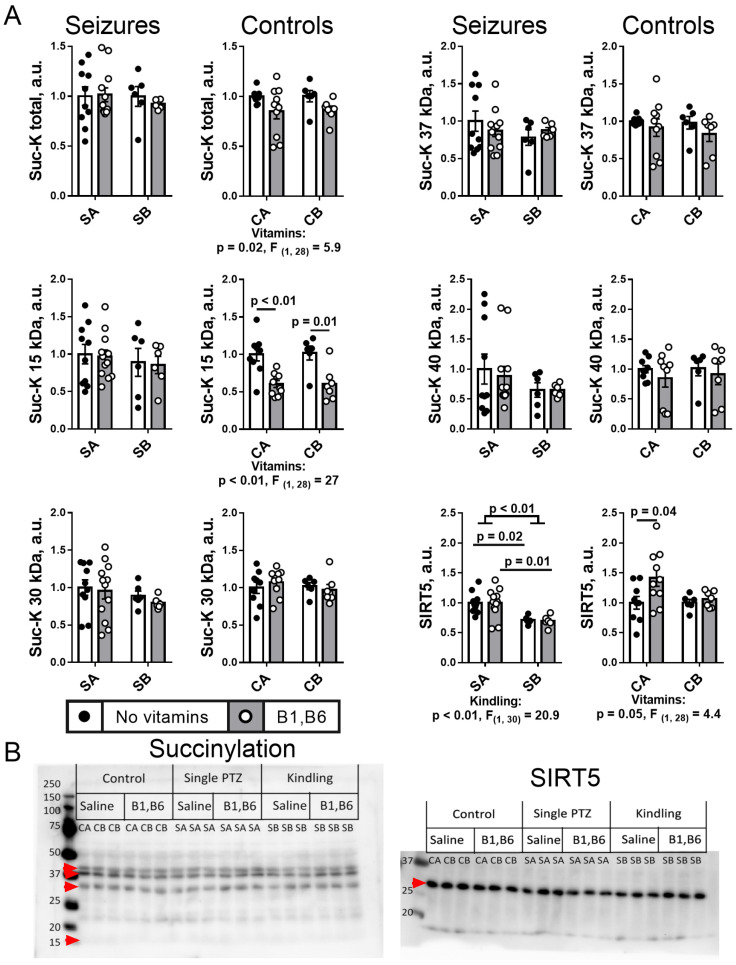
**Effects of the PTZ kindling and vitamins administration on the brain protein succinylation and corresponding deacylase Sirtuin 5** (**A**) **after seizures, in comparison to the effects of the corresponding injections in the control rats. Representative images of the blots are shown in** (**B**)**.** Seizures (S) are induced according to the two protocols shown in Figure 1: SA defines the animals treated by the protocol in Figure 1A, i.e., without the PTZ kindling (n = 10 without vitamins; n = 12 with vitamins); SB defines the animals subjected to the PTZ kindling according to the protocol in Figure 1B (n = 6 without vitamins; n = 6 with vitamins). In the control (C) animals, CA defines those receiving saline instead of vitamins and/or PTZ according to the protocol in Figure 1A (n = 9 without vitamins; n = 10 with vitamins); CB defines the analogous control animals additionally subjected to the repetitive injections of saline instead of PTZ according to the protocol in Figure 1B (n = 6 without vitamins; n = 7 with vitamins). Statistically significant (*p* < 0.05) differences between the experimental groups, determined using the two-way ANOVA with Tukey’s post hoc test, are shown on the graphs. Further, significant factors, determined by the two-way ANOVA, and their statistics, are indicated under the graphs. The assayed parameters are indicated on the Y axes. The amount of succinylated protein (protein lysine residues) and the level of SIRT5 are normalized to total protein and are shown to the levels in the comparison groups without vitamins (CA or SA) (see “Materials and Methods”). The main bands are quantified in addition to the total acylation levels. The original images of the blots are provided as Supplementary Figures S3 and S4.

Unlike acetylation, the protein succinylation is not changed by the PTZ kindling that, however, decreases the expression of the protein desuccinylase SIRT5 (Figure 7). In contrast, a significant decrease in succinylation of 15 kDa proteins including histones, is observed after the vitamins administration to the control animals of both CA ad CB groups (Figure 7). The effect reciprocates the vitamins-induced increase in p53 expression in these animals (Figure 4). In the vitamin-treated CA group, this succinylation decrease is accompanied by a significant (*p* = 0.05) upregulation of the desuccinylase SIRT5, with the effect not observed after the multiple saline injections in CB group (Figure 7).

Thus, PTZ kindling decreases expression of SIRT5. The vitamins-promoted desuccinylation of 15 kDa proteins in the control animals, is blocked by seizures, independent of kindling.

### 2.6. Effects of PTZ Kindling on the Levels of Metabolites

#### 2.6.1. Redox Indicators

**Figure 8:**
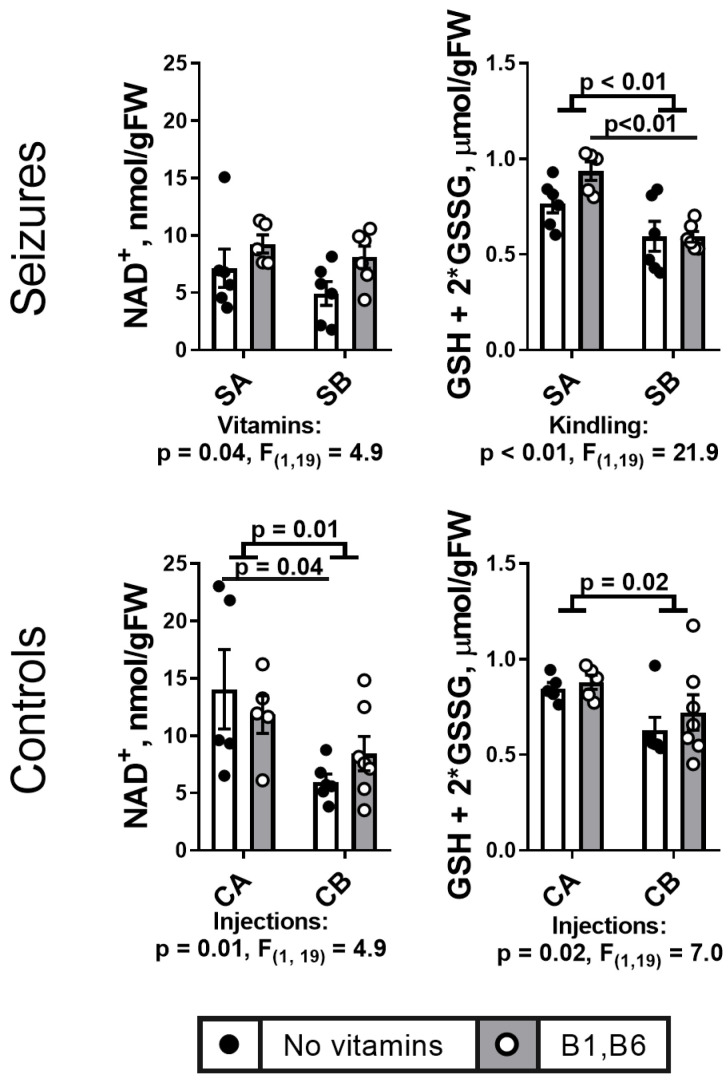
**Effects of the PTZ kindling and vitamins administration on the brain NAD^+^ and total glutathione content after seizures, in comparison to the effects of the corresponding injections in the control rats.** Seizures (S) are induced according to the two protocols shown in Figure 1: SA defines the animals treated by the protocol in Figure 1A, i.e., without the PTZ kindling (n = 5 without vitamins; n = 5 with vitamins); SB defines the animals subjected to the PTZ kindling according to the protocol in Figure 1B (n = 6 without vitamins; n = 6 with vitamins). In the control (C) animals, CA defines those receiving saline instead of vitamins and/or PTZ according to the protocol in Figure 1A (n = 5 without vitamins; n = 5 with vitamins); CB defines the analogous control animals additionally subjected to the repetitive injections of saline instead of PTZ according to the protocol in Figure 1B (n = 6 without vitamins; n = 7 with vitamins). Statistically significant (*p* < 0.05) differences between the experimental groups, determined using the two-way ANOVA with Tukey’s post hoc test, are shown on the graphs. Further, significant factors, determined by the two-way ANOVA, and their statistics, are indicated under the graphs. The assayed parameters are indicated on the Y axes.

A single episode of the PTZ-induced seizures decreases cerebral NAD^+^ level (Figure 8) from 14.1 nmol/g FW (CA group) to 7.1 nmol/g FW (SA group) (*p* = 0.08 according to the Mann–Whitney test). There is no further decrease in NAD^+^ by PTZ kindling (Figure 8, Seizures). In the control animals, the injection factor is significant for the differences between the groups CA ad CB (Figure 8, Controls Panel). In these control groups, NAD^+^ is more significantly decreased without vitamin supplementation, compared to those vitamins-supplemented (Figure 8, Controls). In the PTZ-treated rats, vitamins significantly elevate the NAD^+^ level (Figure 8, Seizures).

In view of our simultaneous quantification of multiple proteins and metabolites, no specific tissue treatment to preserve the glutathione redox state could be employed. In this case, content of total glutathione is chosen as a measure of the brain antioxidant potential, excluding potential contribution of the storage of the oxidatively compromised post-seizure tissue to the glutathione redox status. Figure 8 shows that the total glutathione level is reduced both by the PTZ kindling and multiple saline injections.

#### 2.6.2. Free Amino Acids and Related Compounds

**Figure 9:**
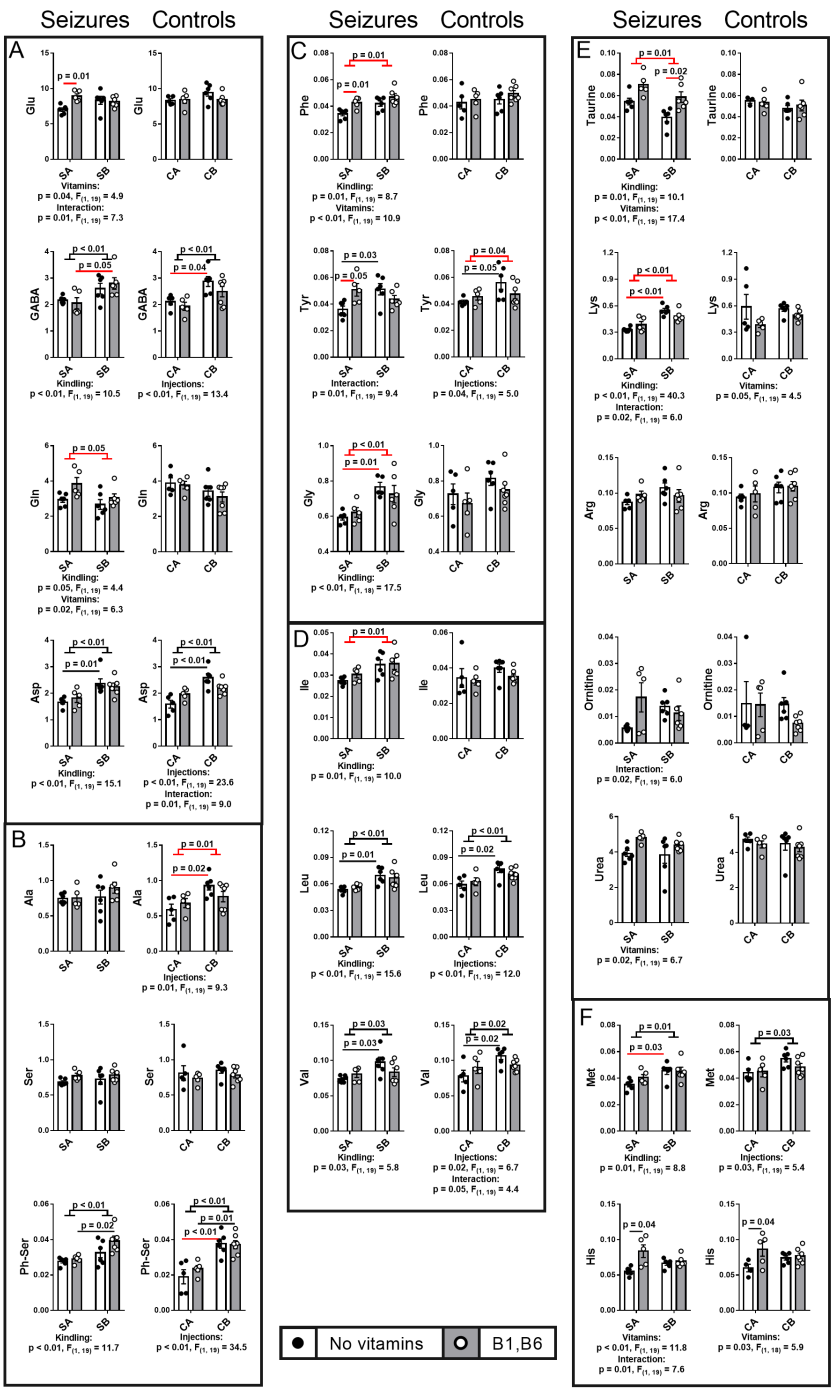
**Effects of the PTZ kindling and vitamins administration on the post-seizures brain levels of amino acids and derivatives, in comparison to the effects of the corresponding injections in the control rats.** The assayed amino acids (in the standard three-letter code) and their derivatives (Ph-Ser is phosphoserine) are indicated on the Y axes. Different effects in the control and seizures groups are indicated with red lines. The assayed metabolites are grouped according to their biological roles discussed in the text. (**A**)—Major excitatory (Glu) and inhibitory (GABA) neurotransmitters and the related abundant amino acids (Glu amidation product Gln and Glu transamination participant Asp). (**B**)—Amino acids related to pyruvate and pyruvate dehydrogenase complex. (**C**)—Neurotransmitter precursors (Phe, Tyr) and neuromodulators (Gly). (**D**)—Branched-chain amino acids supporting glutamate neurotransmission through the transamination. (**E**)—Amino acids and derivatives, related to the redox state. (**F**)—Other. Seizures (S) are induced according to the two protocols shown in Figure 1: SA defines the animals treated by the protocol in Figure 1A, i.e., without the PTZ kindling (n = 5 without vitamins; n = 5 with vitamins); SB defines the animals subjected to the PTZ kindling according to the protocol in Figure 1B (n = 6 without vitamins; n = 6 with vitamins). In the control (C) animals, CA defines those receiving saline instead of vitamins and/or PTZ according to the protocol in Figure 1A (n = 5 without vitamins; n = 5 with vitamins); CB defines the analogous control animals additionally subjected to the repetitive injections of saline instead of PTZ according to the protocol in Figure 1B (n = 6 without vitamins; n = 7 with vitamins). Statistically significant (*p* < 0.05) differences between the experimental groups, determined using the two-way ANOVA with Tukey’s post hoc test, are shown on the graphs. Further, significant factors, determined by the two-way ANOVA, and their statistics, are indicated under the graphs.

Figure 9A shows the major amino acids neurotransmitters and their closest metabolic partners. While glutamate level does not change in the studied groups, its amidation product glutamine is decreased by PTZ kindling only (Figure 9A). The levels of the glutamate decarboxylation product GABA and the glutamate transamination co-product aspartate are increased by both the PTZ kindling and multiple saline injections (Figure 9A). These changes confirm the stress-perturbed metabolism of the amino acids in the brain, complementing the data of the enzymatic assays, but do not discriminate specific actions of PTZ or injections (Figure 5). The pyruvate transamination product alanine, however, is affected by multiple injections of saline only, with the effect abrogated by PTZ injections (Figure 9B), similar to the PDHC activity (Figure 5).

Compared to the short-term action of a single PTZ injection, the long-term procedure of PTZ kindling increases the neurotransmitter precursor phenylalanine, neuromodulator glycine (Figure 9C), and the branched-chain amino isoleucine supporting glutamate neurotransmission via the glutamate-regenerating transamination (Figure 9D). The PTZ kindling decreases taurine (Figure 9E)—a redox-active molecule with cytoprotective properties [55,56]. From the amino acids related to NO-dependent signaling, only lysine shows significant increase by the PTZ kindling.

The administration of vitamins B1 and B6 regulating activities of the amino-acids-related enzymes (Figure 5A,B), modifies the cerebral metabolism of amino acids (Figure 9). In many cases, stronger effects of the vitamins are observed in the animals with single PTZ-induced seizures than after the PTZ kindling. In particular, compared to the single episode of seizures without vitamins, the vitamins administration increases the levels of glutamate (Figure 9A), phenylalanine and tyrosine upon the single seizures (Figure 9C). In the PTZ-kindled group (SB) the vitamins increase the level of taurine (Figure 9E). In other cases, administration of vitamins has minor opposite effects in the SA and SB or CA and CB groups, obvious from decreased statistical significance of the kindling and/or injection stress effects (e.g., tyrosine, glycine, lysine, Figure 9).

Thus, specific PTZ- or injection-stress-induced changes in the brain levels of the amino acids neurotransmitters, their precursors and neuromodulators (Figure 9) complement the changes in the involved enzymes (Figure 5). Administration of the vitamins B1 and B6, providing the coenzymes for some of these enzymes, affect the levels of the brain amino acids, with many of the effects dependent on the animal state. In particular, the vitamins prevent the kindling-induced changes in a number of amino acids by differently affecting the two seizures groups (SA and SB).

### 2.7. Administration of Vitamins B1 and B6 Affects Correlations between the Assessed Parameters in the Post-Seizures and Control Rats

#### 2.7.1. Vitamins Regulate the Seizures-Induced Metabolic and Physiological Changes

To elucidate the combined effect of the B1 and B6 vitamins administration, the correlations between the most relevant biochemical and physiological parameters are analyzed in the pooled seizure groups (SA + SB) without (Table 1, top right) and with (Table 1, bottom left) the vitamins. Remarkably, in the seizure groups (Table 1, top right), a positive correlation between the levels of p53 and neurotoxic glutamate is observed. Although less significant (*p* = 0.07), this correlation is also observed in the vitamins-treated rats. This finding is consistent with the leading role of the seizures-associated glutamate neurotoxicity in the post-seizures p53 induction.

The seizures-relevant effects of the B1 and B6 vitamins are implied by a number of correlations that strongly change in the animals undergoing seizures without the vitamins administration, compared to those with the vitamins. These include the correlations of p53 with the ECG parameters RMSSD and SI, characterizing the autonomous heart regulation; correlations of the seizures duration with physiological parameter and the activity of GDH which in the brain functions in the direction of oxidative deamination of glutamate [57]; correlation of the seizures severity with the brain levels of GABA. All these correlations are observed in the animals without the vitamins supplementation, disappearing after the supplementation. In contrast, the vitamins-supplemented group exhibits a positive correlation of the OGDHC activity with the seizures latency (Table 1, bottom left), absent in the animals undergoing seizures without the vitamins. As a result, the correlation analysis reveals an interplay between the behavioral/ECG changes and the seizures duration, with the vitamins abrogating these relationships along with affecting the correlations of the enzymes (OGDHC, GDH) and metabolites (Glu, GABA), involved in the Glu/GABA balance.

#### 2.7.2. Regulation of Metabolism and Physiology by the Vitamins Administration in the Control Rats

In control rats treated with injections of saline instead of PTZ, a negative correlation of p53 with RMSSD is preserved, but the positive correlation of p53 with SI is not observed (Supplementary Table S1, top right). Thus, unlike the correlation of p53 with RMSSD, the correlation of p53 with SI is specifically related to the post-seizures state. This is consistent with the SI correlation to the seizures duration (Table 1, top right).

Negative correlations of GABA with grooming and rearing, consistent with the GABA action as an inhibitory neurotransmitter, are observed along with a positive correlation of the GABA levels and the GDH activity in the control rats (Supplementary Table S1, top right). Remarkably, in the vitamins-treated control rats, this GABA-GDH correlation disappears. Simultaneously, grooming and rearing become correlated with the activity of OGDHC (Supplementary Table S1, bottom left). The correlation analysis thus indicates that the behavioral effects of the vitamins are mediated by OGDHC, consistent with the essential role of this thiamine-diphosphate-dependent complex in determining the glutamate flux through different pathways [58].

As a result, the correlation analysis reveals that vitamins B1 and B6 affect metabolic relationships between the components supporting the Glu/GABA balance both in the control animals and those exposed to seizures.

## 3. Discussion

In this work, we characterize the multitude of the PTZ-kindling-induced changes in the rat pathophysiology and brain biochemistry. The strength of epileptic seizures, the behavioral and ECG parameters the day after the seizures are measured along with assaying a set of enzymes critical for the brain metabolism of glucose, glutamate and GABA. The set includes the multienzyme complexes of pyruvate dehydrogenase (PDHC) and 2-oxoglutarate dehydrogenase (OGDHC), GDH, GS, transaminases of aspartate (GOT), alanine (GPT) and GABA (GABAT), and the enzymes providing the transaminases with their coenzyme pyridoxal-5′-phosphate, i.e., PLK and PNPO. Finding the correspondence between the biochemical and pathophysiological parameters helps to reveal the brain metabolic pathways with the pathophysiological significance, that is required to discover novel pharmacological targets.

To reveal the changes induced by the PTZ kindling, we compare the parameters of the PTZ-induced seizures and the seizures-affected brain metabolism in the animals undergoing seizures without and after the kindling. The effects are considered to be specifically induced by the PTZ kindling, if they are not observed after the multiple saline injections mimicking the kindling procedure. In this way, we separate the effects of the PTZ kindling from the stress effects in response to multiple injections, required for the kindling. As a result of this analysis, one may conclude that most of the changes in the B6-dependent enzymes are induced by the injection stress, that also affects the B1-dependent enzymes with their network partners (Figure 5). However, the latter enzyme group is specifically affected by the PTZ kindling, either explicitly (OGDHC), or by decreased reactivity to the injection stress (PDHC, MDH, ME, GS), or by the different response to vitamins (GDH).

**Figure 10:**
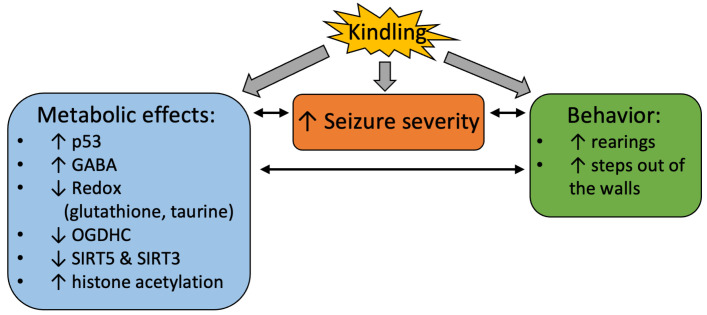
**Interconnected effects of the PTZ kindling on seizures, metabolism and behavior in the seizures-exposed rats.** The arrows near the parameters indicate up- or downregulation of the parameter by kindling.

Remarkably, the PTZ kindling decreases the activity of the rate-limiting enzyme complex of the TCA cycle, OGDHC (Figure 5 and Figure 10), known to be critical for the brain function through its involvement in both the energy and Glu/GABA metabolism [58,59,60]. A wealth of evidence, including the patients’ data, indicate that OGDHC is strongly involved with glutamate excitotoxicity and associated production of reactive oxygen species (ROS) [61,62,63]. On the other hand, in different systems, OGDHC function and/or its saturation with the coenzyme thiamine diphosphate, the diphosphorylated vitamin B1, is known to be tightly linked to p53-dependent regulation of redox metabolism [49,50,64,65,66]. Additionally, in our epilepsy model, PTZ kindling increases expression of p53 (Figure 4 and Figure 10) concomitantly with downregulation of the OGDHC function (Figure 5 and Figure 10), both changes accompanying an increase in the severity of seizures after the kindling (Figure 2 and Figure 10). Thus, repeated challenge of the balance of Glu/GABA neurotransmission by PTZ blockade of the GABA-A receptors deteriorates seizures simultaneously with the increased expression of p53 and decreased flux through the TCA-cycle-limiting OGDHC, where the neurotoxic glutamate may be utilized [63]. Transcriptional control, induced by the PTZ kindling, is evident not only from the p53 upregulation, but also from the decreased expressions of SIRT3 and SIRT5 (Figure 6, Figure 7 and Figure 10), and the kindling-increased acetylation of the brain proteins with high mobility in the SDS-gel electrophoresis, inherent in histones (apparent molecular mass 15 kDa, Figure 6 and Figure 10).

The kindling-induced p53 upregulation at the level of the protein expression (Figure 4 and Figure 10) may be added by the functional activation of p53 through the simultaneous decrease in the SIRT5 expression (Figure 7 and Figure 10), as desuccinylation of Lys120 in p53 by SIRT5 suppresses the transcriptional activity of p53 upon the DNA damage response [67]. This mechanism may explain the neuroprotective action of SIRT5 known from earlier studies [68]. Deacetylation of p53 by SIRT3 may also be involved in the p53-dependent metabolic control. This regulation is more studied in aging and cancer [69]. In particular, SIRT3 suppresses the p53-mediated ferroptosis in cancer cells [70]. Thus, in addition to the increased expression of p53 (Figure 4 and Figure 10) the decreases in expression of SIRT3 and SIRT5 after the PTZ kindling (Figure 6, Figure 7 and Figure 10), may stimulate the p53 transcriptional activity after the kindling through post-translational acylations. Although regulatory phosphorylation of p53 at Ser392 in the protein C-terminus does not exhibit a significant change after the PTZ kindling, the ratio of the phosphorylated p53 to its expression level does not change (Figure 4), suggesting the p53 phosphorylation to increase proportionally to the protein expression.

In the control groups, we observe a stress response to multiple injections of saline, that is clearly different from the effects of the PTZ kindling in the seizure groups. Mimicking the PTZ kindling protocol, but not the PTZ action as such, the injections of saline result in changes in the brain enzymatic activities linked to the pyruvate levels (Figure 5). Obviously, the stress affects the pyruvate flux distribution through the TCA cycle and affiliated pathways. PDHC activity is increased along with a decrease in the activity of MDH. As less oxaloacetate—a product of the MDH reaction—may thus be condensed with the PDHC-produced acetyl-CoA, the observed changes in the PDHC and MDH activities manifest a regulation directed to decreased consumption of the acetyl-CoA in the TCA cycle. Moreover, production of pyruvate by the oxaloacetate-decarboxylating malic enzyme increases, supporting the stress-elevated PDHC activity by a higher pyruvate supply. The higher supply is also evident from the increase in the pyruvate transamination sibling alanine (Figure 9B). Finally, increased activity of the brain GS may point to increased degradation of the amino acids through the TCA cycle [71]. Feeding the cycle at different entry points, the amino acids degradation may compensate for the insufficient acetyl-CoA flux from PDHC to the TCA cycle. However, the degradation produces an excess of ammonia, whose utilization by GS not only attenuates the ammonia toxicity to the brain, but also preserves the nitrogen in the form of glutamine. Alternative to the oxidation in the TCA cycle, the increased production of acetyl-CoA by PDHC, supported by increased activity of malic enzyme, may be used for the increased biosynthetic needs, e.g., of acetylcholine. Remarkably, the adjustments of the brain enzymatic activities in response to the injection stress do not result in perturbed acylation of the brain proteins (Figure 6 and Figure 7), as occurs upon the PTZ kindling (Figure 6 and Figure 7) or a short-term PDHC inhibition [51,72].

The PTZ kindling affects mitochondrial metabolism not only in our experimental design where the effects of the kindling are characterized in the animals exposed to seizures, but also in other studies, where the effects of PTZ kindling are revealed by comparison to the control group [73]. Coincidence of the data in different models stresses the role of mitochondria in the kindling-induced changes. In particular, compared to the control animals, daily administration of PTZ to rats elevates expression of nitric oxide synthase (NOS) in cerebellar neurons and increases the NO generation, with the NO content positively correlated to the severity of seizures [74]. Accordingly, administration of arginine is shown to potentiate the PTZ-induced seizures, whereas NOS inhibitors or NOS knockout reduces the seizures [26,74,75]. The release of NO is observed upon stimulation of glutamatergic neurons [76,77], resulting in predominance of excitatory over inhibitory signals, similarly to the action of PTZ. Here, we do not measure NO, but assess potential changes in NO metabolism after the PTZ kindling through the levels of relevant amino acids (Figure 9E). Such changes are implicated by increased level of lysine in the PTZ-kindled rats vs. the rats undergoing seizures without the kindling (Figure 9E). In the reaction with arginine, producing ornithine, lysine generates an alternative source of NO, homoarginine (reviewed in [78]). Using our data (Figure 9D) for the pair-wise comparison of the kindled and control rats by the Mann–Whitney test, the levels of lysine (Figure 9D, SA vs. CA, *p* = 0.03) and ornithine (Figure 9D, SA vs. CA, *p* = 0.05) are reduced after a single seizure episode, and re-established after the PTZ kindling. Thus, our data add to the independent studies suggesting that effects of seizures on NO metabolism in the brain depend on the PTZ kindling.

It is worth noting that, compared to a single seizure episode, the PTZ kindling increases the level of glycine (Figure 9C), whose brain metabolism and signaling are known to be involved into epileptic seizures. For instance, microperfusion of glycine in hippocampus of rats with changed membrane distribution of the NMDA receptors induces seizures in 75% of the rats [79]. Glycine levels are known to be increased in a rat model of the kindling by electrical stimulation of amygdala [80]. The number of glycine-binding sites of the NMDA receptors is increased one month after the kindling [81]. Inhibition of these strychnine-insensitive glycine receptors by 7-chlorokynurenic acid suppresses the kindling effect [82], as does a downregulation of hippocampal glycine transporter 1, whose expression is increased in patients and rodent models of temporal lobe epilepsy [83]. The pro-convulsive action of glycine in these models is in good accord with our results, where the PTZ-kindling-induced increase in the brain glycine level, compared to a single PTZ-induced seizure (Figure 9C), accompanies an increase in the severity of seizures (Figure 2A). Remarkably, Figure 9C shows that the kindling-induced elevation in the brain glycine levels (*p* = 0.01, Tukey’s post hoc test, in the rats receiving no vitamins), is not significant after the supplementation of vitamins B1 and B6 (*p* > 0.05, Tukey’s post hoc test). Stimulation of the B6-dependent glycine decarboxylase may be involved in the effect. Significance of this enzyme for the brain levels of glycine, and the pro-convulsive action of glycine are evident from epileptic seizures induced by impairments of glycine decarboxylase causing glycine accumulation [84].

The PTZ kindling also perturbs antioxidant potential of the brain. Although accurate determination of redox state of glutathione in our tissue samples is complicated by its oxidation into GSSG during storage [85], our finding of the kindling-induced decrease in total glutathione (Figure 8) confirms the perturbed antioxidant power of the brain after the seizures. The antioxidant taurine is also decreased after the kindling (Figure 9E). The kindling-induced decrease in taurine, observed in our model, has also been observed in another study, along with increase in GABA [86], observed by us as well (Figure 9A).

Similar to increasing the seizure severity score (Figure 2), the PTZ kindling procedure induces an increase in rearings and steps out of the walls. The link between these behavioral parameters and seizures is substantiated by significant positive correlations of both rearings and steps out of the walls with the duration of clonic seizures in the PTZ-treated animals (Supplementary Table S1). Remarkably, after the seizures, the two parameters do not correlate to each other, as they do, independent of the vitamins administration, in the control animals. However, the vitamin administration to the seizure groups re-establishes the correlation between the rearings and steps out of the walls, testifying to a normalizing effect of the vitamins on the brain metabolism and behavior. It may be speculated, that the seizures-associated increases in rearings and steps out of the walls are related to the PTZ blocking the inhibitory GABA-A receptors [14]. It is also worth noting in this regard that several reports indicate an increase in hippocampal neurogenesis in PTZ-kindled animals [87,88], supposed to play a role in the pathophysiology or represent a compensatory process in epilepsy [89]. This effect of PTZ kindling on neurogenesis may contribute to the increases in the abovementioned parameters of exploratory activity, observed in our PTZ-kindling model (Figure 2).

Thus, our study reveals significant metabolic changes in the post-seizures brain exposed to the PTZ kindling, which may be alleviated by the administration of vitamins B1 and B6.

## 4. Materials and Methods

Animal experimental employ the reagents from the following manufacturers: thiamine (#36020.02)—«Serva», Heidelberg, Germany; pyridoxal (#A0960)—«PanReac AppliChem», Barcelona, Spain, NAD^+^ (#1013)—“Gerbu”, Heidelberg, Germany; malate dehydrogenase—«Reanal», Budapest, Hungary; other reagents for biochemical procedures as well as PTZ (#P6500) —«Sigma-Aldrich», St. Louis, MO, USA. Pyridoxine-5′-phosphate is synthesized from pyridoxal-5′-phosphate (#P9255) («Sigma-Aldrich», St. Louis, MO, USA) as described previously [90] and kindly provided by Dr. Martino di Salvo (Sapienza University, Rome, Italy). The solutions are prepared in Milli-Q deionized water, the salts used are of the highest purity available.

### 4.1. Animal Models

Our study uses Wistar male rats obtained from the Russian Federation State Research Center Institute of Biomedical Problems RAS (IBMP). The animals are kept under standard conditions in cages with free access to water and food and a light/dark cycle of 12/12 h (the light turned on at 9 a.m.). The adaptation period to the husbandry conditions is two weeks. Animals are distributed randomly between experimental groups. The animal experiments are approved by Bioethics Committee of Lomonosov Moscow State University (protocols 69-o from 9 June 2016 for single PTZ-induced seizure model and 139-a from 11 November 2021 for the PTZ kindling model). In both models, the age of the animals at the time of the seizure induction by PTZ is 10–11 weeks (weight 320 ± 20 g).

#### 4.1.1. Model of Single Episode of PTZ-Induced Seizures

Single episode of seizures (animal group SA) is induced in the 10–11-week-old male rats by intraperitoneal administration of PTZ in saline at 25 mg/kg dose according to the protocol in Figure 1A.

After PTZ administration, the severity of seizures is visually assessed according to the modified Racine scale developed for the employed protocol of PTZ administration [91] (Table 2) for 15 min in individual cages (OpenScience, Moscow, Russia).

The total time of the seizure observation is 45 min.

During the visual assessment, there are no more than 4 rats in individual cages (2 with PTZ and 2 with saline (0.9% NaCl) injections). This arrangement allows one to observe each of the rats. Scores are registered every minute of the observation, and an epileptic seizure of maximum score is noted for the given minute of observation. If the stages 4–5 on the modified Racine scale (tonic or tonic-clonic seizures, Table 2) do not develop within 15 min, PTZ is re-injected at the same dose of 25 mg/kg. The procedure is repeated no more than three times, and the total PTZ dose thus does not exceed 75 mg/kg. The “seizure severity” score is calculated as the average score of the severity of an epileptic seizure over the entire observation period from the moment of administration of the first PTZ dose.

#### 4.1.2. Model of PTZ-Induced Seizures after the PTZ Kindling

As we aim at comparison of the consequences of the acute PTZ-induced seizures occurring at the same age, the rats entering the kindling experiment are younger (Figure 1B), than those in the single PTZ-induced seizure episode (Figure 1A). Taking into account 19 days required for the PTZ kindling according to our previously published protocol [29], the kindling is started with the 7–8-week-old male rats of 140 ± 10 g. This kindling model of epilepsy (animal group SB) is shown in Figure 1B. The scheme is selected to minimize mortality, simultaneously achieving a strong manifestation of the clonic seizures according to the Racine scale (see Table 2). The rats are injected PTZ i.p. at an originally sub-convulsive dose of 37.5 mg/kg 3 times a week for 3 weeks, resulting in the total 9 injections of PTZ and its total dose of 337.5 mg/kg during the PTZ kindling. Each of the injections is followed by the visual assessment of the animals according to Table 2, as described above. A week after the last injection of 37.5 mg/kg PTZ in the kindling procedure, the PTZ administration to induce seizures is performed exactly as in the model of a single seizure episode (Figure 1). 24 h post-seizures the rats are physiologically tested and sacrificed by decapitation, as shown in the flow-chart in Figure 1. The procedure is performed exactly as described before [94].

#### 4.1.3. Administration of Vitamins

Vitamins B1 (thiamine, 100 mg per kg body weight) and B6 (pyridoxal, 100 mg per kg body weight) are administered intraperitoneally twice, first time 24 h before the first administration of 25 mg/kg PTZ and second time after completion of a 45 min follow-up of the PTZ-induced seizures, either in the single seizure model or following PTZ kindling (Figure 1). This vitamin regimen takes into account the results of previous studies on the potential protective effect of high doses of vitamins before [95] or after [65] the exposure to stress, when increased availability of vitamins may provide, respectively, the better stabilization or normalization of the metabolic state [96]. The injections with equivalent volumes of saline (0.9% NaCl) instead of the vitamins are given to the reference group.

According to the formula recommended by the US Food and Drug Administration (http://www.fda.gov/downloads/Drugs/GuidanceComplianceRegulatoryInformation/Guidances/ucm078932.pdf (accessed on 5 May 2023), the doses of vitamins B1 and B6 used in this study on rats (100 mg/kg each of the vitamins per day) correspond to a dose of 16 mg/kg, or 1 g for an average weight of 60 kg, in humans. These doses are within the interval of the doses used in the vitamin therapy [46,48,97,98,99].

#### 4.1.4. Animal Survival

The data on the total number of animals in our experiments and their survival is summarized in Table 3.

#### 4.1.5. Control Animals to Reveal the Injections-Related Stress Effects

As the kindling protocol is associated with multiple injections, the effects of PTZ kindling are discriminated from the effects of injections without the substances in the control animals CA (protocol of Figure 1A) or CB (protocol of Figure 1B) receiving saline (0.9% NaCl) or saline with vitamins according to the group assignment, at the times and in the volumes, equal to those of the PTZ and/or vitamins solutions.

### 4.2. Assessment of Physiological Parmeters after Seizures and in the Corresponding Control Groups

To assess the spontaneous behavior of animals in an unfamiliar environment, the «Open Field» test («OpenScience», Moscow, Russia) is used [100]. To quantify the animal behavior, the animals are tested for 3 min in complete silence under the light of a 15 W red lamp as described before [46,52]. Locomotor activity is estimated by the number of line crossings. Anxiety level is determined, registering defecation acts, duration of freezing, the duration and number of grooming acts. Exploratory activity is assessed by the number of rearing acts, steps out of the walls and entries to the center.

ECG is recorded for 3 min using non-invasive electrodes as previously described [46]. Balance of the heart autonomous regulation is assessed by the following parameters of ECG: an average R-R-interval (R-R interval, ms), standard deviation of an average R-R (SD, ms), a range of R-R interval values, i.e., a difference between the maximal and minimal values (dX, ms), root mean square of successive differences in R-R intervals (RMSSD, ms), and stress index (SI, arbitrary units).

### 4.3. Preparation of Homogenates of the Rat Cerebral Cortex

The procedure is performed exactly as described before [94]. After the animal decapitation, the brain is excised and transferred onto ice, where the cerebral cortex is separated for freezing in liquid nitrogen within 60–90 s after decapitation. The cortices are stored at −70 °C. Homogenization of the tissue and sonication of homogenates is carried out according to the previously published protocol [94].

### 4.4. Measurement of Enzymatic Activities

The activities of oxoglutarate dehydrogenase complex (OGDHC), pyruvate dehydrogenase complex (PDHC), glutamate dehydrogenase (GDH), malate dehydrogenase (MDH), glutamine synthetase (GS) and NADP^+^-dependent malic enzyme (ME) are measured in cerebral cortex homogenates as described previously [71,101]. Prior to the activity assays, except for that of GS, the homogenates are sonicated, following by the addition of one volume of solubilization buffer (50 mM Tris-HCl, pH 7.4, 600 mM NaCl, 4 mM EDTA, 1% sodium deoxycholate, and 4% NP-40) to the three volumes of sonicated homogenate as described before [94].

Activity of the GABA transaminase (GABAT) is measured by the published assay [102]. Briefly, 10 µL of sonicated cerebral cortex homogenate per microplate well is used and the reaction is started by addition of reaction mixture comprising 100 mM Tris-HCl, pH 8.1, with 2 mM 2-oxoglutarate, 1 mM NAD^+^, 20 mM 2-mercaptoethanol, and 3 mM GABA.

The activities of GOT and glutamate pyruvate transaminase (GPT) are measured using a coupled reaction with MDH or LDH, respectively. Briefly, the GPT is measured using 2 µL of sonicated homogenate added per well, and the reaction is started by addition of reaction mixture comprising 100 mM NaKHPO_4_ buffer with 0.1 mM NADH, 2 mM 2-oxoglutarate, 40 mM DL-alanine and 4 U/mL LDH, pH 7.4. Similarly, GOT is measured using 3 µL of sonicated homogenate diluted 25 times in 50 mM MOPS buffer pH 7.0, added per well. The reaction is started by addition of reaction mixture comprising 100 mM NaKHPO_4_ buffer with 0.1 mM NADH, 2 mM 2-oxoglutarate, 40 mM DL-aspartate and 5 µg/mL MDH, pH 7.4.

The activities of pyridoxal kinase (PLK) and pyridoxine-5′-phosphate oxidase (PNPO) are measured in black 96-well microplates using the fluorescence mode of a CLARIOstar Plus plate reader (BMG LABTECH, Ortenberg, Germany) according to the published protocol [46]. All other activities are assayed spectrophotometrically in transparent 96-well microplates, measuring absorbance at 500 (PDHC), 540 (GS) or 340 (all the rest) nm by a Sunrise plate reader (Tecan, Grödig, Austria).

### 4.5. Western Blotting

Cerebral cortex homogenates are diluted in Laemmli buffer and subjected to SDS-PAGE. The resulting gels are used for the assessment of protein expression via 2,2,2-tricholoroethanol staining as described before [65]. Total protein succinylation is assessed using primary antibodies #PTM-1151 from PTM-Biolabs (Chicago, IL, USA), expression of sirtuins 3 and 5 and total protein acetylation—#5490, #8782 and #9841, respectively, from Cell Signaling Technology (Danvers, MA, USA). These primary antibodies were used at 1:2000 dilution. The expression of p53 protein and its phosphorylated form (pS392-p53) were assessed using primary antibodies #MA5-12453 and #44-640G from Invitrogen (Waltham, MA, USA) at 1:350 and 1:500 dilution, respectively. Secondary HRP-conjugated anti-rabbit antibody #7074 from Cell Signaling Technology (Danvers, MA, USA) is used in 1:3000 dilution. Chemiluminescence is detected using ChemiDoc MP Imager (Bio-Rad, Hercules, CA, USA) and processed in Image Lab software v. 6.0.1 (Bio-Rad, Hercules, CA, USA). The intensities of the quantified parameters are normalized to total protein in the lane and shown as a ratio to the parameter intensities in the comparison groups, i.e., to the averaged values of animals receiving no vitamins in control (C) or seizure (S) groups from protocol A (Figure 1). Protein intensities from different membranes are compared across all the membranes through normalization to the intensities of several common samples repeated on all the membranes. The original images of membranes and gels with visualized protein bands are presented in Supplementary Figures S1–S5.

### 4.6. Preparation of Tissue Extracts and Quantifications of Metabolites

Methanol-acetate extraction of metabolites from the rat cerebral cortex and quantification of its amino acids and urea are performed as described before [103,104]. Briefly, frozen cerebral cortices are homogenized in ice-cold methanol, then acetic acid solution is added and the proteins are precipitated. The sodium-citrate buffer system with a Hitachi L-8800 amino acid analyzer is used according to the published protocol [103]. NAD^+^ quantification in methanol-acetic extracts of rat cortices is performed using recombinant formate dehydrogenase according to the previously published protocol [105]. Reduced (GSH) and oxidized (GSSG) glutathione content is measured using fluorometric assay with o-phthalaldehyde [106] as described before [101]. Since no specific techniques have been employed to minimize glutathione oxidation during the storage of extracts, only changes in the total glutathione (GSH + 2 × GSSG) are analyzed.

### 4.7. Statistical Analysis and Data Presentation

Data are analyzed using the GraphPad Prism 7.0 software (GraphPad Software Inc., La Jolla, CA, USA). Individual values of the parameters for each animal, their mean and SEM are shown on the graphs. D’Agostino-Pearson omnibus normality testing and ROUT test for outliers are used. Statistical significance of differences upon comparison of four experimental groups differing in two factors such as “vitamins” and “kindling” or “vitamins” and “injections”, is assessed using two-way analysis of variance (ANOVA) with Tukey’s post hoc test. In some additional comparisons of specific two groups, mentioned in the data discussion, the Mann–Whitney U-test is employed. The statistical methods are indicated in the text and/or figure legends. The statistical significances at *p* ≤ 0.05 are shown on the graphs. The ANOVA results for the significant (*p* ≤ 0.05) factors and their interaction are also shown below the figures together with the corresponding F-statistics.

## 5. Conclusions

The PTZ-kindling-induced changes in the rat pathophysiology and brain biochemistry are characterized, revealing significant relationships between the post-seizures brain metabolism and behavior with the seizures parameters. In particular, expression of p53 in the post-seizures brain correlates to the brain level of glutamate and stress index of the heart autonomous regulation. The correlations are absent in the control animal group and in the animals exposed to seizures after administration of vitamins B1 and B6, pointing to the alleviation of the post-seizures effects by the vitamins. The seizures-modified behavior correlates to the seizures duration. Absence of the correlations in the vitamins-treated group substantiates the positive action of vitamins on the post-seizures brain. Indeed, the vitamins are shown to affect the metabolic enzymes and levels of the amino acids neurotransmitters and their precursors, dependent on the seizures and kindling exposures. The vitamins also increase the brain levels of NAD^+^ in the animals exposed to seizures. The sensitivity of the relationships between the post-seizure behavior and seizures parameters to vitamins B1 and B6, characterized in this work, provides a basis for future human studies of the neuroprotective effects of the vitamins in repeated epileptic seizures.

## Figures and Tables

**Table 1:** Correlations between the parameters of seizures and selected post-seizures biochemical and physiological parameters in dependence on the vitamins administration. The correlations for the rats subjected to seizures without (top right, n = 12–16) or with the administration of vitamins B1 and B6 (bottom left, n = 11–18) are built for the combined groups SA (seizures, model A, see Figure 1) and SB (seizures, model B, see Figure 1). Every cell contains a Spearman correlation coefficient (above) and its *p*-value (below). The cells with significant (*p* < 0.05) *p*-values are colored in red or blue, corresponding to the positive or negative correlation coefficients.

	No Vitamins	GDH	OGDHC	p53	Glu	GABA	Grooming Acts	Rearing Acts	Steps Out	RMSSD	SI	Seizure Latency	Seizure Severity	Seizure Duration
Vitamins														
GDH			−0.42 0.10	−0.26 0.47	0.26 0.42	0.34 0.28	0.31 0.23	0.20 0.45	0.80 0.00	0.08 0.78	−0.38 0.16	−0.11 0.69	0.30 0.26	0.58 0.02
OGDHC		0.11 0.66		−0.42 0.23	−0.34 0.28	−0.32 0.31	−0.04 0.89	0.11 0.68	−0.40 0.13	0.28 0.31	−0.11 0.69	0.18 0.51	−0.23 0.39	−0.21 0.45
p53		−0.21 0.54	−0.26 0.43		0.79 0.05	0.18 0.71	−0.08 0.83	−0.63 0.06	−0.46 0.18	−0.80 0.01	0.77 0.02	0.52 0.13	0.41 0.25	−0.36 0.30
Glu		−0.07 0.84	0.37 0.26	0.75 0.07		0.38 0.22	0.19 0.54	−0.17 0.59	0.04 0.90	−0.45 0.17	0.37 0.26	0.45 0.15	0.36 0.25	−0.03 0.94
GABA		0.33 0.33	−0.41 0.21	0.43 0.35	0.33 0.33		0.48 0.11	0.35 0.27	0.55 0.07	0.15 0.67	−0.11 0.75	−0.16 0.61	0.59 0.05	0.23 0.49
Grooming acts		0.27 0.29	−0.33 0.18	0.01 0.98	−0.13 0.72	0.15 0.65		0.53 0.04	0.44 0.09	0.20 0.46	−0.12 0.67	0.14 0.61	0.07 0.81	0.57 0.02
Rearing acts		0.33 0.18	−0.34 0.17	0.27 0.42	−0.26 0.44	0.57 0.07	0.19 0.44		0.42 0.11	0.34 0.22	−0.33 0.22	−0.28 0.29	−0.12 0.67	0.53 0.03
Steps out		0.35 0.15	−0.30 0.22	0.20 0.55	−0.36 0.27	0.40 0.22	0.45 0.06	0.66 0.00		0.37 0.18	−0.37 0.17	−0.25 0.35	0.16 0.54	0.59 0.01
RMSSD		−0.33 0.18	−0.21 0.41	0.15 0.67	0.14 0.69	0.25 0.47	−0.16 0.53	0.27 0.27	0.25 0.32		−0.70 0.00	−0.17 0.53	−0.28 0.32	0.23 0.41
SI		0.25 0.31	0.37 0.13	−0.36 0.27	−0.10 0.78	−0.40 0.22	0.22 0.38	−0.27 0.28	−0.23 0.36	−0.70 0.00		0.32 0.25	−0.05 0.85	−0.59 0.02
Seizure latency		0.29 0.24	0.69 0.00	−0.22 0.51	0.24 0.47	−0.07 0.83	−0.32 0.19	−0.05 0.84	−0.22 0.38	−0.05 0.83	0.19 0.46		0.37 0.15	−0.27 0.32
Seizure severity		−0.16 0.53	−0.20 0.42	0.16 0.63	−0.49 0.13	0.33 0.33	−0.09 0.72	0.53 0.02	0.45 0.06	0.41 0.09	−0.51 0.03	0.20 0.42		0.15 0.60
Seizure duration		−0.43 0.08	−0.21 0.40	−0.20 0.73	−0.42 0.20	−0.33 0.33	0.28 0.27	−0.23 0.35	0.07 0.77	−0.05 0.83	0.30 0.23	−0.34 0.17	−0.08 0.75	

**Table 2:** Modified Racine scale for visual assessment of the severity of PTZ-induced seizures in rats [6,92,93].

Score	Behavioral Manifestations of Seizures
0	Normal behavior, no abnormality
1	immobilization, lying on belly
2	head nodding, facial, forelimb or hindlimb myoclonus
3	myoclonic twitches, continuous whole-body myoclonus, tail held up stiffly
4	clonic rearing, bilateral clonic seizure, falling down on a side
5	tonic-clonic seizure, falling down on back, wild rushing and jumping

**Table 3:** Initial (n_0_) and final (n) number of animals, with their survival in each group. The seizures are induced by PTZ without (A) or with (B) PTZ kindling.

Seizure Model	Group	n_0_	n	Survival, %
A. Single seizure episode (up to 7 injections)	Seizure induction	10	10	100
	Seizure induction with B1,B6	12	12	100
	B1,B6 (saline and vitamin injections)	10	10	100
	Control (saline injections)	9	9	100
B. Seizure episode after PTZ kindling (up to 16 injections)	Kindling + seizure induction	6 *	6	100
	Kindling + seizure induction with B1,B6	8 *	6	75
	B1,B6 (saline and vitamin injections)	7	7	100
	Control (saline injections)	6	6	100

* A total of 3 out of 17 rats in the kindling group died during kindling period (two after the 5th PTZ injection and one after the 8th one).

## Data Availability

The data presented in this study are available in this article (summarized in figures and Tables, including Supplementary Data). The raw data are available on request from the corresponding author.

## Supplementary Materials

The following supporting information can be downloaded at: https://www.mdpi.com/article/10.3390/ijms241512405/s1.

## Abbreviations

PTZ	pentylenetetrazole
OGDHC	2-oxoglutarate dehydrogenase complex
PDHC	pyruvate dehydrogenase complex
GDH	glutamate dehydrogenase
MDH	malate dehydrogenase
PLK	pyridoxal kinase
PNPO	pyridoxal 5′-phosphate oxidase;
GS	glutamine synthetase
ME	malic enzyme
GOT	glutamate-oxaloacetate transaminase
GPT	glutamate-pyruvate transaminase
GABA	gamma-aminobutyric acid
GABAT	GABA transaminase
NOS	nitric oxide synthase
