# Supplementary material for: Pentylenetetrazole-Induced Seizures Are Increased after Kindling, Exhibiting Vitamin-Responsive Correlations to the Post-Seizures Behavior, Amino Acids Metabolism and Key Metabolic Regulators in the Rat Brain
Source: Int J Mol Sci. 2023 Aug 3;24(15):12405. doi: 10.3390/ijms241512405 (PMC10418815; doi:10.3390/ijms241512405)
Supplement: Supplementary file 1 [file ijms-24-12405-s001.zip › ijms-2543052-supplementary.pdf]

Pentylenetetrazole-induced seizures are increased after kindling, exhibiting vitamin-responsive correlations to the post-seizures behavior, amino acids metabolism and key metabolic regulators in the rat brain

Vasily A. Aleshin, Anastasia V. Graf, Artem V. Artiukhov, Alexander L. Ksenofontov, Lev G. Zavileyskiy, Maria V. Maslova, Victoria I. Bunik

Supplementary Data

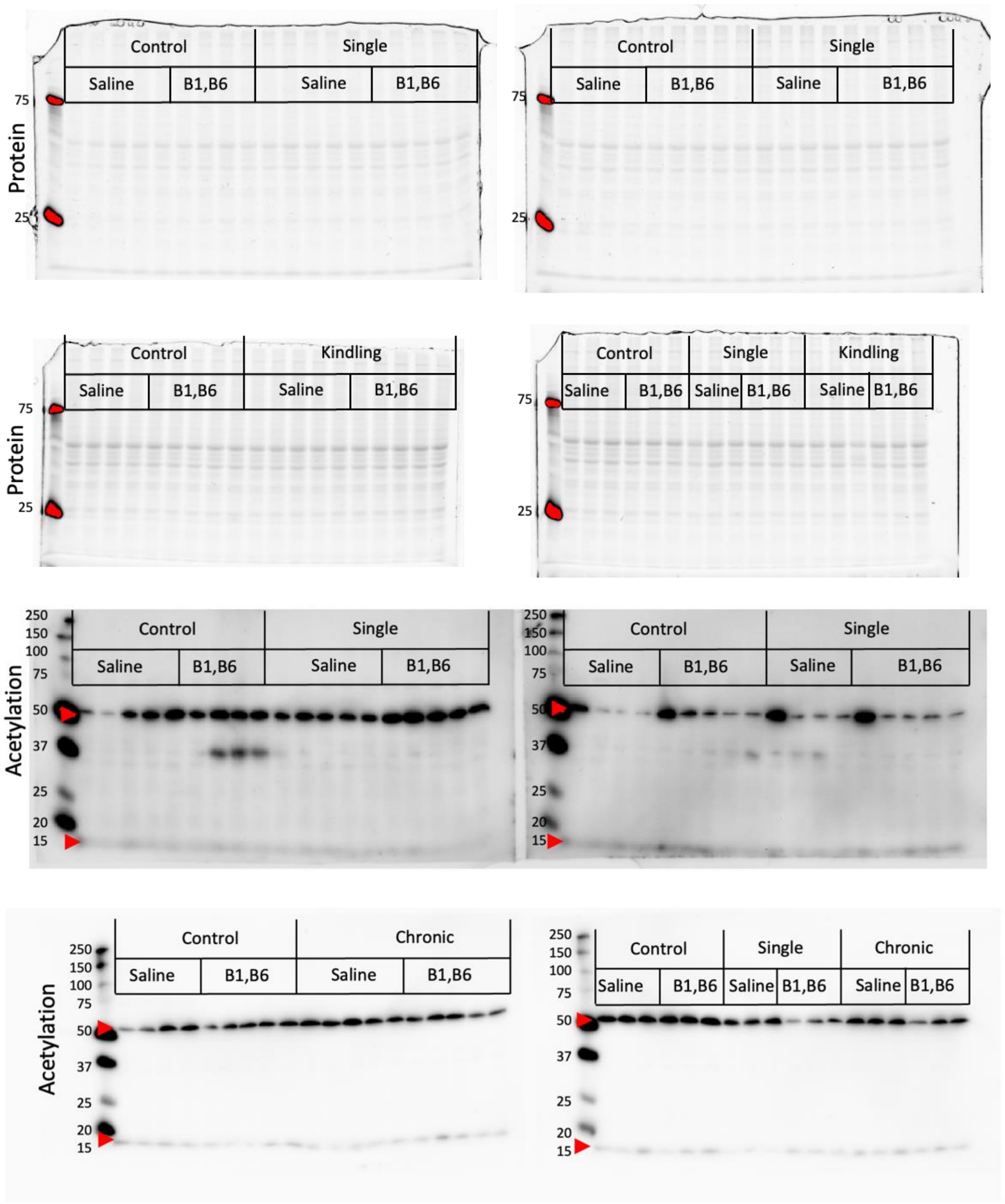

Figure S1. Four gels (upper panel) represent total protein content corresponding to Western blots for protein acetylation (acetylated lysine residues) (lower panel).

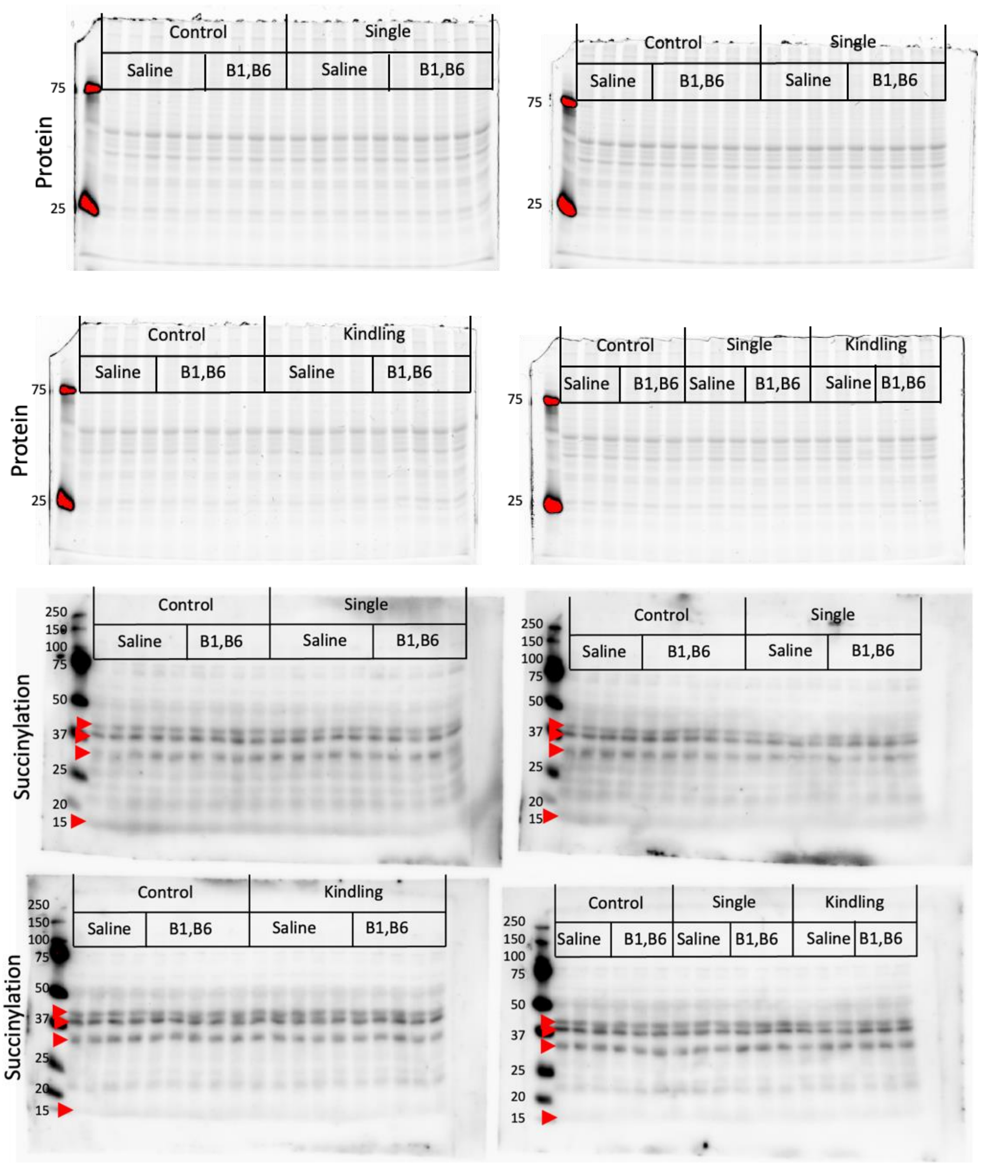

Figure S2. Four gels (upper panel) represent total protein content corresponding to Western blots for protein succinylation (succinylated lysine residues) (lower panel).

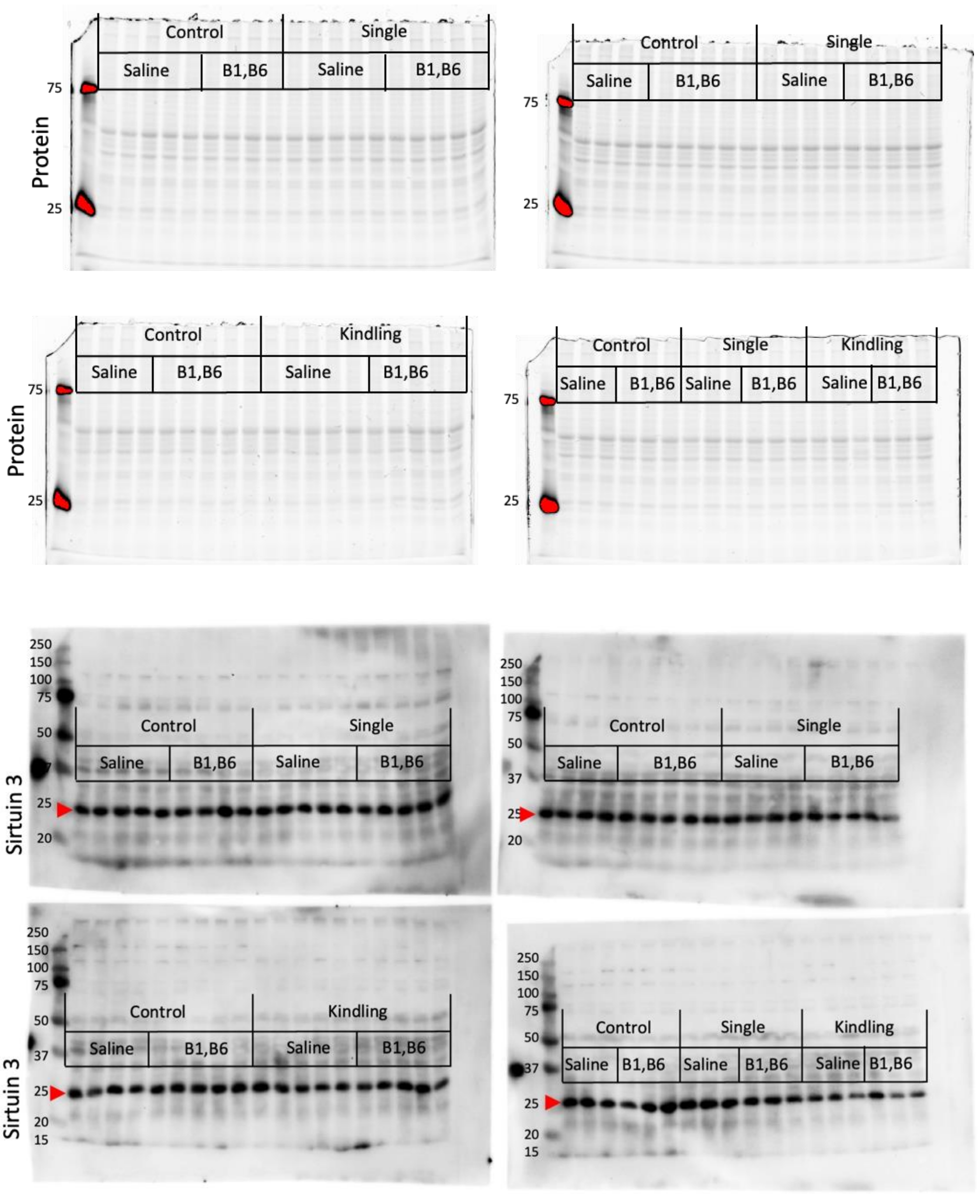

Figure S3. Four gels (upper panel) represent total protein content corresponding to Western blots for Sirtuin 3 (lower panel).

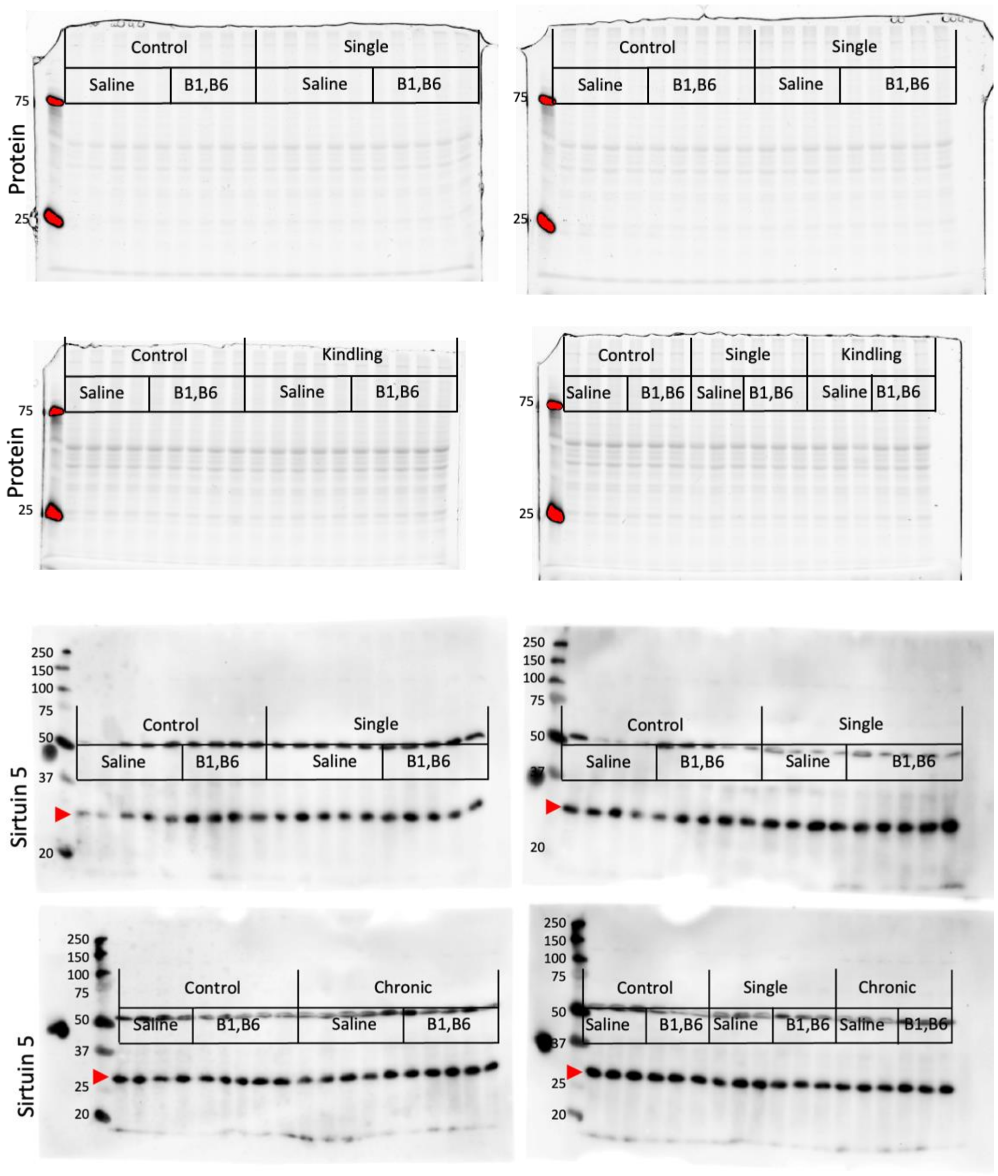

Figure S4. Four gels (upper panel) represent total protein content corresponding to Western blots for Sirtuin 5 (lower panel).

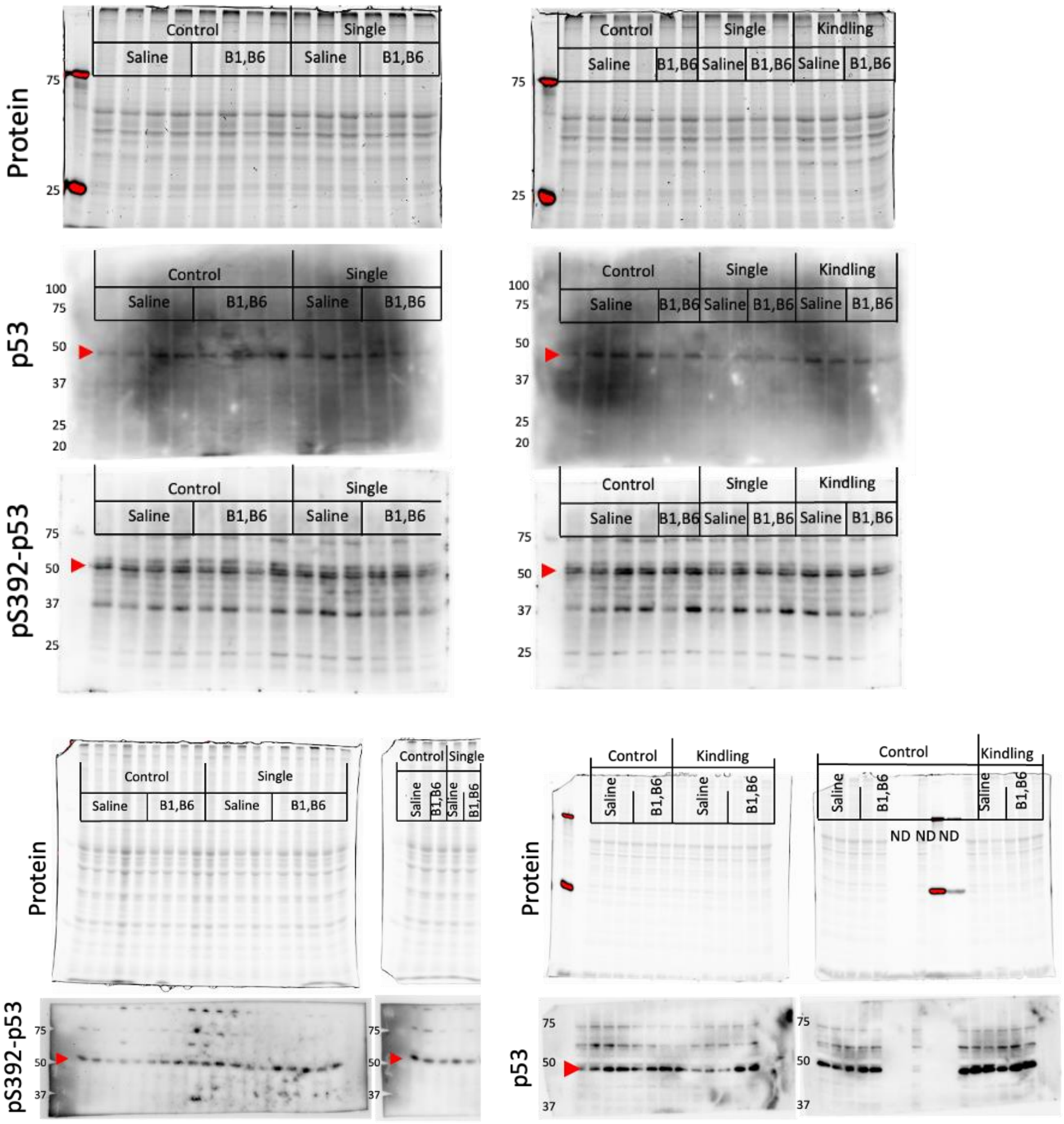

Figure S5. Gels and corresponding Western blots represent total protein content and p53 or pS392-p53 (p53 phosphorylated at Ser392). The parameters are indicated on the figure. Colorimetric MW markers were used, their positions are indicated with digits.

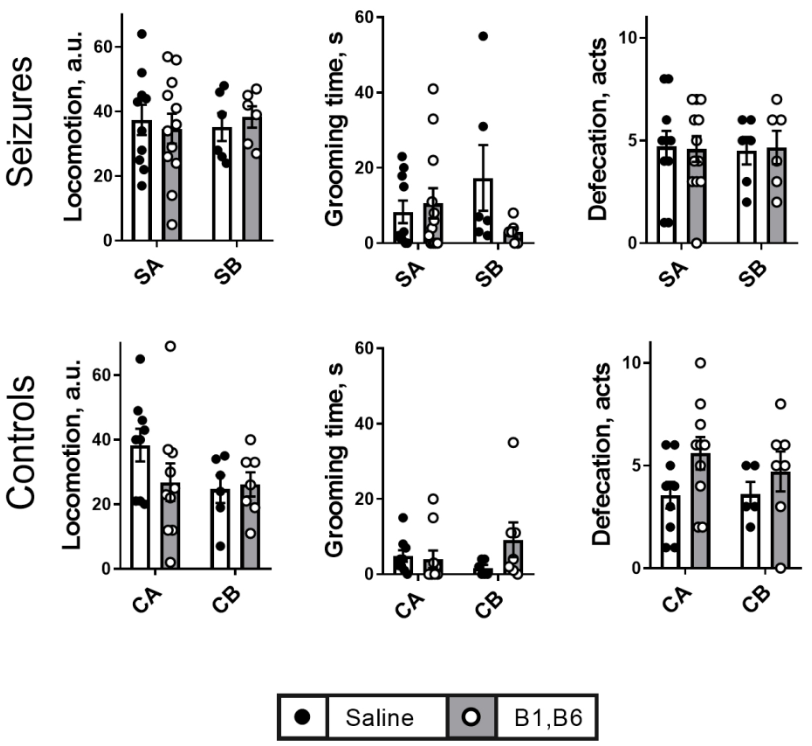

Figure S6. Additional behavioral parameters assessing the effect of the PTZ kindling and vitamins. The experimental groups are designated as in Figure 2. The assayed parameters are indicated on the Y axes.

Table S1. Correlations between selected biochemical and physiological parameters in the control rats, and their dependence on the vitamins administration. The correlations in the control rats without (top right, n=11-15) and with the administration of vitamins B1 and B6 (bottom left, n=12-17) are built for the combined control groups CA (control, model A, see Figure 1A) and CB (control, model B, see Figure 1B). Every cell contains a Spearman correlation coefficient (above) with its p-value (below). The cells with significant ($p<0.05$) p-values are colored in red or blue, corresponding to the positive or negative correlation coefficients.

No vitamins / Vitamins	GDH	OGDHC	p53	Glu	GABA	Grooming acts	Rearing acts	Steps out	RMSSD	SI
GDH		-0.31<br>0.26	0.06<br>0.86	0.54<br>0.09	0.93<br>0.00	-0.49<br>0.06	-0.47<br>0.08	-0.25<br>0.37	-0.42<br>0.12	0.64<br>0.01
OGDHC	0.27<br>0.32		0.04<br>0.92	-0.12<br>0.73	-0.58<br>0.06	-0.01<br>0.97	0.45<br>0.09	0.31<br>0.25	0.28<br>0.31	-0.23<br>0.40
p53	0.54<br>0.07	0.48<br>0.12		-0.04<br>0.96	-0.18<br>0.71	0.15<br>0.65	-0.36<br>0.27	-0.39<br>0.23	-0.65<br>0.04	0.31<br>0.36
Glu	0.10<br>0.77	-0.20<br>0.56	0.50<br>0.22		0.48<br>0.13	-0.55<br>0.09	-0.35<br>0.29	-0.48<br>0.14	0.04<br>0.92	0.17<br>0.61
GABA	0.10<br>0.75	-0.51<br>0.11	-0.14<br>0.75	0.37<br>0.24		-0.63<br>0.04	-0.63<br>0.04	-0.31<br>0.35	-0.64<br>0.04	0.63<br>0.04
Grooming acts	0.07<br>0.80	-0.56<br>0.03	-0.25<br>0.43	-0.17<br>0.59	0.12<br>0.71		0.07<br>0.79	0.01<br>0.97	0.38<br>0.17	0.03<br>0.92
Rearing acts	-0.44<br>0.08	-0.58<br>0.02	-0.29<br>0.36	-0.39<br>0.21	-0.30<br>0.34	0.24<br>0.36		0.57<br>0.03	0.35<br>0.19	-0.53<br>0.04
Steps out	-0.29<br>0.27	-0.28<br>0.29	-0.13<br>0.68	0.27<br>0.39	0.15<br>0.65	0.22<br>0.40	0.54<br>0.03		0.37<br>0.17	-0.36<br>0.19
RMSSD	0.22<br>0.39	0.24<br>0.37	0.13<br>0.70	0.14<br>0.67	-0.13<br>0.68	-0.09<br>0.72	-0.26<br>0.31	-0.43<br>0.08		-0.51<br>0.05
SI	0.03<br>0.91	0.05<br>0.86	-0.01<br>0.97	-0.08<br>0.80	-0.37<br>0.24	-0.02<br>0.93	0.07<br>0.79	0.01<br>0.97	-0.63<br>0.01	
